# Amplification of *CDK4* and *MDM2*: a detailed study of a high-risk neuroblastoma subgroup

**DOI:** 10.1038/s41598-022-16455-1

**Published:** 2022-07-20

**Authors:** Angela Martinez-Monleon, Hanna Kryh Öberg, Jennie Gaarder, Ana P. Berbegall, Niloufar Javanmardi, Anna Djos, Marek Ussowicz, Sabine Taschner-Mandl, Inge M. Ambros, Ingrid Øra, Bengt Sandstedt, Klaus Beiske, Ruth Ladenstein, Rosa Noguera, Peter F. Ambros, Lena Gordon Murkes, Gustaf Ljungman, Per Kogner, Susanne Fransson, Tommy Martinsson

**Affiliations:** 1 Department of Laboratory Medicine, University of Gothenburg, Box 445, 405 30 Gothenburg, Sweden; 2 Department of Pathology, Faculty of Medicine and Dentistry, University of Valencia, Valencia, Spain; 3 Department of Pediatric Bone Marrow Transplantation, Oncology and Hematology, Wroclaw Medical University, 50-556 Wrocław, Poland; 4 St. Anna Children’s Cancer Research Institute (CCRI), Vienna, Austria; 5 Department of Pediatric Oncology and Hematology, Clinical Sciences, Lund University, Lund, Sweden; 6 Childhood Cancer Research Unit, Karolinska Institutet, Stockholm, Sweden; 7 Institute of Clinical Medicine, University of Oslo, Oslo, Norway; 8 Department of Pediatric Radiology, Astrid Lindgren Children’s Hospital, Karolinska University Hospital, Stockholm, Sweden; 9 Department of Women’s and Children’s Health, Children’s University Hospital, University of Uppsala, Uppsala, Sweden; 10 Department of Women’s and Children’s Health, Karolinska Institutet, Stockholm, Sweden

**Keywords:** Biomarkers, Cancer, Cancer genetics, Cancer genomics, Cancer therapy, Oncology, Paediatric cancer

## Abstract

In neuroblastoma, *MYCN* amplification and 11q-deletion are important, although incomplete, markers of high-risk disease. It is therefore relevant to characterize additional alterations that can function as prognostic and/or predictive markers. Using SNP-microarrays, a group of neuroblastoma patients showing amplification of one or multiple 12q loci was identified. Two loci containing *CDK4* and *MDM2* were commonly co-amplified, although amplification of either locus in the absence of the other was observed. Pharmacological inhibition of CDK4/6 with ribociclib or abemaciclib decreased proliferation in a broad set of neuroblastoma cell lines, including *CDK4/MDM2*-amplified, whereas MDM2 inhibition by Nutlin-3a was only effective in p53^wild-type^ cells. Combined CDK4/MDM2 targeting had an additive effect in p53^wild-type^ cell lines, while no or negative additive effect was observed in p53^mutated^ cells. Most 12q-amplified primary tumors were of abdominal origin, including those of intrarenal origin initially suspected of being Wilms’ tumor. An atypical metastatic pattern was also observed with low degree of bone marrow involvement, favoring other sites such as the lungs. Here we present detailed biological data of an aggressive neuroblastoma subgroup hallmarked by 12q amplification and atypical clinical presentation for which our in vitro studies indicate that CDK4 and/or MDM2 inhibition also could be beneficial.

## Introduction

Neuroblastoma (NB) is a pediatric cancer that affects 1 in 8000 children and, despite its relative rarity, is a leading cause of cancer-related death in childhood. It derives from immature cells of the sympathetic nervous system originating from the neural crest. The primary tumors commonly manifest in the abdomen along sympathetic ganglia or in the medullary region of the adrenal gland. Over half of these patients already have metastasis at diagnosis, the most common sites being bone marrow (70%), cortical bone (56%), regional and distant lymph nodes (31%), liver (30%), and intracranial and orbital sites (18%), while rarer locations are lung (3%) or central nervous system (0.6%)^1^. The clinical behavior varies greatly, ranging from spontaneous regression or maturation to aggressive tumor growth with fatal outcome despite intensive multimodal therapy. Poor prognosis is especially apparent among patients with high-risk neuroblastoma (HR-NB), with less than 50% 5-year survival^2,3^.

The clinical heterogeneity of this disease is also reflected at the molecular level, at which distinct genomic features of the tumor are critical for stratification and treatment decision. Whereas there is a relative paucity of recurrent single-nucleotide variants (SNVs)^4–6^, persistent copy number alterations (CNAs) are common, so analysis of somatic chromosomal aberrations is crucial^7^. Tumors with good prognosis are generally near triploid and display aneuploidy with whole chromosome gains and losses (i.e., numerical chromosomal aberrations, NCAs). High-risk tumors with poor prognosis are predominantly near di- or tetraploid and are associated with recurrent segmental chromosomal aberrations (SCAs), such as deletion of chromosome arm 11q or amplification of the *MYCN* oncogene (MNA)^7^. Other genomic features linked to adverse outcome include 1p-deletion, 1q-gain, distal 6q-deletion, 19q loss, and 17q-gain^7–11^. Further genomic alterations related to poor prognosis are those associated with telomere maintenance, such as aberrations in *ATRX* or *TERT* genes, as well as genetic alterations in the RAS-MAPK pathway, oncogenic mutations of *ALK*, or low-frequency mutations in *TP53*^12,13^.

MNA is a well-defined biomarker present in 25–30% of all NB, and MNA analysis has been mandatory in clinical practice since the 1980s due to its prognostic and diagnostic value^14,15^. Apart from the *MYCN* locus amplification, a focal amplification of the *ALK* gene has been detected in 1–2% of the tumors, almost exclusively in association with *MYCN* amplification^16^. In addition to *MYCN* and *ALK*, other amplification regions have been described, although they are infrequent, including various chromosome segments such as 7q21, 11q13, and 16q22^10,17^. The oncogenic contribution of these amplicons is still unclear, emphasizing the need for further characterization of these regions. We and others have previously also reported high-grade amplification of chromosomal regions 12q13.3–14.1 and 12q15 in a small subset of tumors, sometimes in combination with gain of the chromosomal region 11q13 that includes the *CCND1* gene encoding Cyclin D^7,10,17–22^. Amplification of chromosome 12 regions and over-expression of both *MDM2* and *CDK4* are frequently detected in lipo- and osteosarcoma, in which this amplification is associated with poor prognosis^23–27^. This has also been reported in other cancers including bladder^28^, breast^29^, brain^30^, and lung^31^ cancers and melanomas^31^. Among the different malignancies displaying 12q amplification, two regions mentioned before located at 12q13.3–14.1 and 12q15 are commonly co-amplified. These loci are centered on two genes with a seminal role in cancer development: cyclin-dependent kinase 4 (*CDK4*), a key regulator of cell cycle progression, and mouse double minute 2 (*MDM2*), a proto-oncogene involved in p53 regulation. However, cases in which these amplicons appear independent of each other have also been reported^23,28,32^.

To further elucidate the extent, frequency, and role of the amplification of chromosomal regions in 12q in NBs, we present a detailed analysis of the genetic and clinical features of this subset of NB cases. Furthermore, through cell-based assays, we demonstrate that the pharmacological inhibition of MDM2 and CDK4 is feasible and that these inhibitors decrease proliferation. Therefore, MDM2 and/or CDK4 inhibition could provide an alternative targeted therapy in this patient group.

## Results

### Analysis of copy number and amplicons

**Figure 1:**
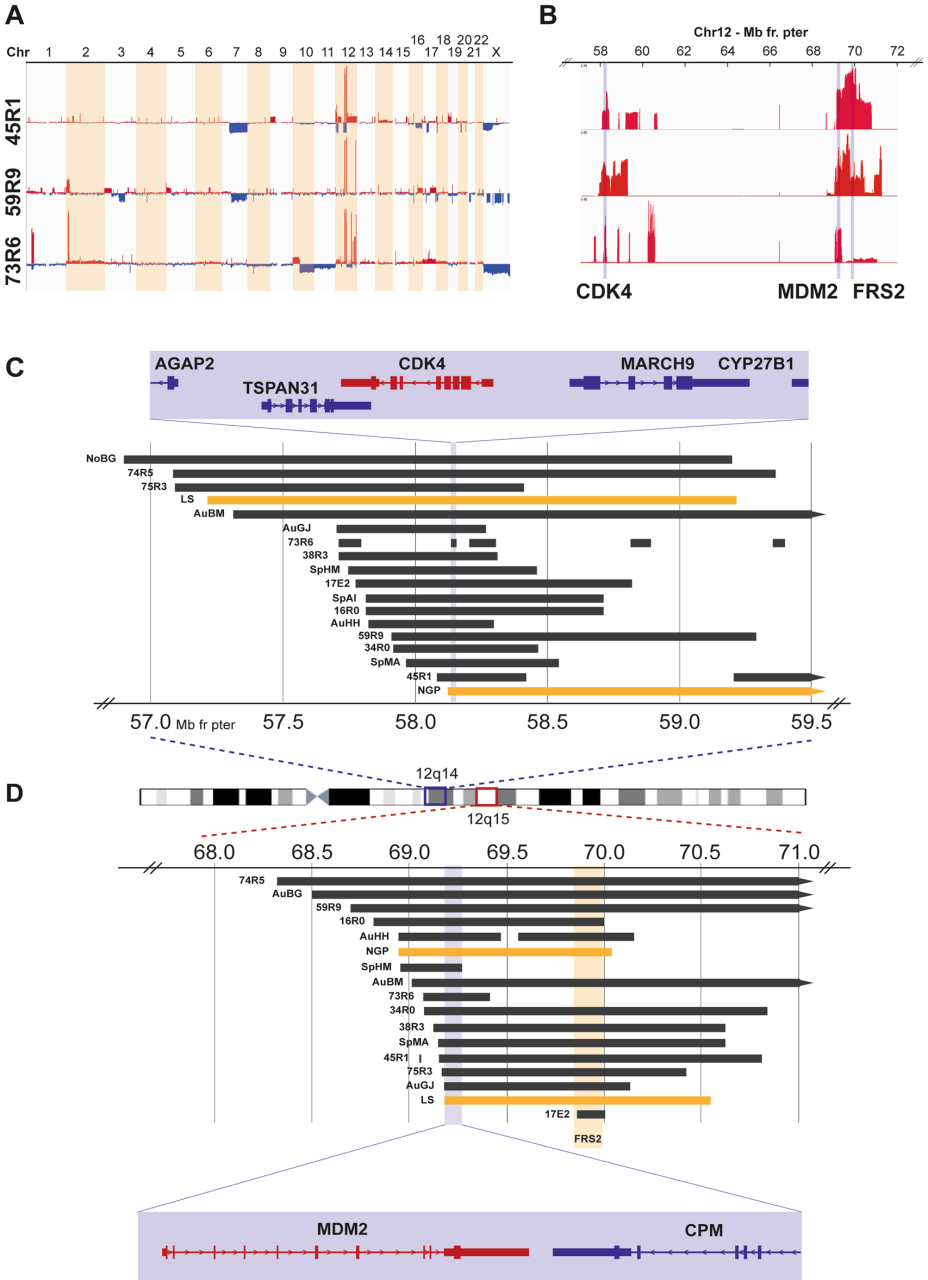
Genomic findings regarding the tumors with 12q amplification. (**A**) Whole-genome copy number profiles generated from WGS for three NB tumors with 12q amplification and (**B**) copy number profiles for chromosome 12, 57–72 Mb from the p-terminal with corresponding amplification peaks. (**C**) Amplified regions at 12q14 in relation to genomic position and genes with the shortest region of overlap (SRO), indicated in blue, based on amplicon boundaries of all primary tumors and cell lines analyzed. (**D**) Amplified regions at 12q15 in relation to genomic position and genes with the SRO indicated in blue.

Among genomic profiles derived from SNP microarrays of NB tumors from 436 Swedish patients, we identified nine patients with tumors displaying structural rearrangements and high-grade amplification involving chromosomal regions in the 12q chromosome arm. Eight additional patients with 12q-amplified NB tumors from Poland, Austria, Norway, and Spain as well as two 12q-amplified cell lines were included in the study. Although the genomic profiles of the individual cases differed in the number of amplified regions in chromosome 12, and in the size and junction sites of these amplicons, two loci in 12q13–14 and 12q15 were consistently co-amplified in 16 out of the 19 NB cases (Fig. 1 and Supplemental Fig. 1).

The most proximal of the two regions (12q14.1) was amplified in 18 out of the 19 cases and centered on the *CDK4* gene. The shortest region of overlap (SRO) of the amplified loci at 12q14.1 was located at Chr12: 58.135–58.270 Mb (defined by 73R6 and AuGJ), harboring the genes *TSPAN31*, *CDK4*, *MARCH9*, *CYP27B1*, *METTL1*, *METTLE21B*, *TSFM*, *AVIL*, and *CTDSP2* (Fig. 1C)*.* The more distal region at 12q15 was amplified in 17 out of the 19 cases, mainly centered on *MDM2*, although one of the 12q15 amplicons started in *FRS2* intron 1 located distally to *MDM2.* The SRO of this more distal amplified region (12q15) was located at Chr12: 69,166–69,270 Mb (defined by AuGJ and SpHM) and contained the genes *NR_073494*, *MDM2*, and *CPM* (partial) (Fig. 1D)*.* The fibroblast growth factor receptor substrate 2 (*FRS2*) gene is located distal to *MDM2* at 69.86–69.97 Mb within the 12q15 chromosomal region. The complete *FRS2* gene was included among the 12q15 amplicons in the tumors of 14 cases. To summarize, co-amplification of *CDK4* and *MDM2* was seen in 15 cases (including the two cell lines), amplification of *CDK4* in the absence of *MDM2* was seen in three cases, and *MDM2* amplification in the absence of *CDK4* was seen in one case (Table 1).

**Table 1:** Study cohort: biological, genetic, and clinical details.

Case	38R3	17E2	16R0	AuBM^a,c^	73R6	SpMA	NoBG	45R1	34R0	59R9^a,b^	AuGJ^a,c^	SpHM	AuHH	74R5	75R3	AuBG	SpAI	LS (cell line)	NGP (cell line)
**Patient information**																			
Gender	F	F	M	M	M	M	M	F	M	M	F	F	F	F	F	M	M	F	M
Age at diagnosis (months)	14	33	58	42	25	111	NA	32	63	63	81	100	87	44	48	26	142	16/36	30
INSS	3?	2A	3	3	2	1	2B	4	4	4	4	4	4	4	NA	4	4	3	4
INRG	L	L	L	L2	L2	L1	L2	M	M	M	M	M	M	M	M	M	M	L	M
Survival outcome	NED	NED	DOD	DOD	DOD	DOD	NA	DOD	DOD	DOD	DOD	DOD	DOD	DOD	AWD	DOD	DOD	DOD	DOD
Survival (months)	186+	337+	26	15	8	28	NA	8	10	67	100	33	30	6	17+	7	14	NA	NA
**Clinical findings**																			
Primary tumor	Abd	Abd	R Ren	Abd	Abd/Ren	Abd	NA	R Ren	Abd/Ren	Abd	Abd	Abd/Ren	Abd	Abd	Abd	Abd	Abd	Abd	NA
Localization of primary	L Adr	Adr	R Adr	L Adr	Adr	Adr	NA	R Adr	Adr	NA	L Adr	Renal	R Adr	Adr	Adr	R Adr	Adr	NA	NA
Clinical presentation	AT	OMS	AP	AT	NA	NA	NA	AP	AP	NA	AP	NA	AP			fatigue	NA	NA	NA
Wilms’ tumor like	+	−	+	−	+	−	NA	+	+	−	+	−	−	−	−	−	−	NA	NA
Disease course					Relapse		Relapse			Relapse		Relapse					Relapse		
**Metastases**																			
Bone marrow	L	L	L	L	+^d^	L	NA^d^	−	−	−	−	−	+	+	+	+	−	L	+
Bone	L	L	L	L	L	L	NA^d^	−	−	−	−	−	+	+	+	+	−	L	−
Lung	L	L	L	L	L	L	NA^d^	+	+	+	+	+	+	−	−	−	−	L	+
Other sites	L	L	L	L	L	L	NA^d^	NA	NA	+	+	+	−	+	−	+	+	L	NA
Ext. into vena cava	−	−	+	−	NA	−	NA^d^	+	+	NA	+	−	+	−	−	−	−	NA	−
**Biology**																			
Genomic profile	MNA	OS	OS	hetMNA	MNA	17q	11q	OS	17q	17q	hetMNA	17q	OS	MNA	OS	MNA	11q	MNA	MNA
MYCN amplification	+	−	−	Het^e^	+	−	−	−	−	2p-gain	Het^e^	−	−	+	−	+	−	+	+
11q-deletion	−	−	−	−	−	+(distal)	+	−	−	−	−	−	wcl	−	−	− (UPD11)	+	−	+
1p-deletion	−	−	−	−	Chromo	+	−	−	−	−	−	−	−	−	−	−	−	1p/1q imbalance	1p/1q imbalance
17q-gain	−	wcg	−	+	wcg	+	+	−	+	+	wcg	+	−	+	wcg	wcg	+	+	+
11q/CCND1 gain	−	−	+	−	−	−	−	−	+	−	wcg	−	−	−	11q-gain	−	+	amplified	−
CDK4 amplification	+	+	+	+	+	+	+	+	+	+	+	+	+	+	+	−	+	+	+
MDM2 amplification	+	−	+	+	+	+	−	+	+	+	+	+	+	+	+	+	−	+	+
FRS2 amplification	+	−	+	+	−	+	−	+	+	+	+	−	+	+	+	+	−	+	+
ATRX/TERT	−	−	TERT SV	ATRX del	−	−	ATRX del	−	ATRX (D937N)	−	−	ATRX (E1327X)	ATRX (S2147R)	−	−	−	TERT SV	−	TERT SV
Massive parallel sequencing	−	Exome	−	−	WGS		−	WGS	Exome	WGS	Exome		Exome	−	−	−		−	−

*F* Female, *M* Male, *L* Localized, *M* Metastasized, *INSS* International Neuroblastoma Staging System, *INRG* International Neuroblastoma Risk Group, *NED* No evidence of disease, *AWD* Alive with disease, *DOD* Dead of disease, *NA* information not available, *Abd* Abdominal, *Adr* Adrenal, left (L) or right (R), *Ren* Renal, *AP* Abdominal pain, *AT* Abdominal tumor without pain or other symptoms, *OMS* Opsoclonus-myoclonus syndrome, *MNA* MYCN amplification, *hetMNA* Heterogeneous MYCN amplification, *17q* 17q-gain without MNA or 11q-deletion, *OS* Other structural, *Chromo* Chromothripsis, *wcg* Whole-chromosome gain, *wcl* Whole-chromosome loss, *SV* Structural variant, *del* Deletion. ^a^Patients previously described; ^b^Fransson et al., Sci Rep 2020; ^c^Bogen et al., Int J Can 2016; ^d^Initial localized disease, metastatic spreading occurring after two local relapses; ^e^According to iFISH.

Furthermore, among this group of 12q-amplified NBs, concomitantly amplified loci included *MYCN* (seven cases including the two cell lines), multiple loci in 1p (one case), *ALK* (one case), and *CCND1* (cell line LS) (Supplementary Fig. 1). In addition to the *CCND1* amplification seen in the cell line, CNAs involving *CCND1* were observed in four cases in which three tumors displayed a small focal gain and one tumor a large 11q gain. Segmental gain of 17q was seen in ten cases, whereas whole chromosome 17 gain was seen in five cases (Supplementary Fig. 1).

### Expression analysis and survival analysis

We hypothesized that the amplified regions could harbor one or a few oncogenes that drive tumor progression and that the oncogenic effect is dose dependent, meaning that amplification leads to increased mRNA levels. Among the genes analyzed in the two 12q regions, 12 had over 20-fold higher expression in the 12q-amplified group compared with the 12q non-amplified tumors, whereas an additional 12 genes had at least two-fold higher expression compared with the non-amplified tumors. A total of 25 out of 28 analyzed genes displayed statistically significant differences in expression levels (Table 2).

**Table 2:** Gene expression analysis of genes in amplified regions.

	Gene	12 amp vs. others				
		Transcription start point	Presence in amplified tumors	Fold change	*p*-value	No. of samples with determined ct value
12q14.1	KIF5A	57,943,846	3/5	**3.7**	**3.6E−06**	35
	PIP4K2C	57,984,941	3/5	**7.9**	**1.2E−06**	32
	DTX3	57,998,603	4/5	**15.7**	**1.4E−09**	33
	ARHGEF25	58,003,962	4/5	**4**	**1.3E−05**	35
	SLC26A10	58,013,692	4/5	**5.3**	**0.005**	29
	B4GALNT1	58,019,550	4/5	**5.8**	**1.7E−07**	33
	OS9	58,087,737	5/5	**23**	**1.4E−17**	36
	AGAP2	58,118,075	5/5	**2.5**	0.378	27
	TSPAN31	58,138,783	5/5	**28.5**	**2.2E−13**	32
	CDK4	58,141,509	5/5	**17.4**	**5.8E−09**	36
	MARCH9	58,148,880	5/5	**26**	**1.5E−18**	33
	CYP27B1	58,156,116	5/5	**36**	**2.8E−11**	26
	METTL1^a^	58,162,350	5/5	–	–	–
	METTL21B	58,166,382	5/5	**20.8**	**2.4E−07**	21
	TSFM	58,176,527	5/5	**22.7**	**4.3E−17**	35
	AVIL	58,191,159	5/5	**40.2**	**8.5E−10**	29
	CTDSP2	58,213,709	5/5	**24.1**	**7.9E−12**	34
	XRCC6BP1	58,335,444	5/5	**28.5**	**3.3E−06**	33
	LRIG3	59,265,936	1/5	1.7	0.699	28
	SLC16A7	60,083,125	0/5	1.9	0.728	23
12q15	SLC35E3	69,139,935	4/5	**10.8**	**2.5E−07**	32
	MDM2	69,201,970	4/5	**21.9**	**9.30E−10**	33
	CPM	69,244,955	4/5	1.1	0,88	29
	CPSF6	69,633,316	4/5	**12**	**9.0E−06**	34
	LYZ	69,742,133	4/5	1.5	0.703	35
	YEATS4	69,753,531	4/5	**5.6**	**9.9E−04**	30
	FRS2 ^probe #1^ FRS2 ^probe #2^	69,864,128	4/5	**26.8 41.0**	**4,6E−06 5.97E−04**	30
						27
	CCT2	69,979,207	5/5	**24.6**	**9.4E−06**	34
	LRRC10^a^	70,002,344	5/5	–	–	–
	BEST3^b^	70,047,388	4/5	–	–	1
	RAB3IP	70,132,630	3/5	**9.3**	**1.6E−05**	32

Bold letters indicate a fold change greater than 2 or a p-value less than 0.05. ^a^No probes available; ^b^Determinable Ct value in only one sample.

Kaplan–Meier analysis of the Swedish cohort (*n* = 36) indicates that high expression levels of *CDK4*, *CYP27B1*, and *TSFM* are associated with worse outcome, although not at a statistically significant level. Analysis of two datasets consisting of 88 and 498 NB tumors, publicly available in the R2 database, indicates that high expression of these three genes is associated with low overall survival, with the strongest association between adverse outcome and high expression levels for *CDK4* (*p* = 9.1 × 10^–8^ and *p* = 2 × 10^–20^, respectively) (Supplementary Table 2).

Among the five 12q-amplified tumors included for gene expression analysis, one (i.e., 17E2) did not include *MDM2* in the 12q15 amplicon. This sample displayed *MDM2* expression at levels similar to those of non-amplified NB tumors with different genomic profiles (e.g., numerical only, MNA, and 11q-deleted) (Supplementary Fig. 3).

### Clinical features

**Figure 2:**
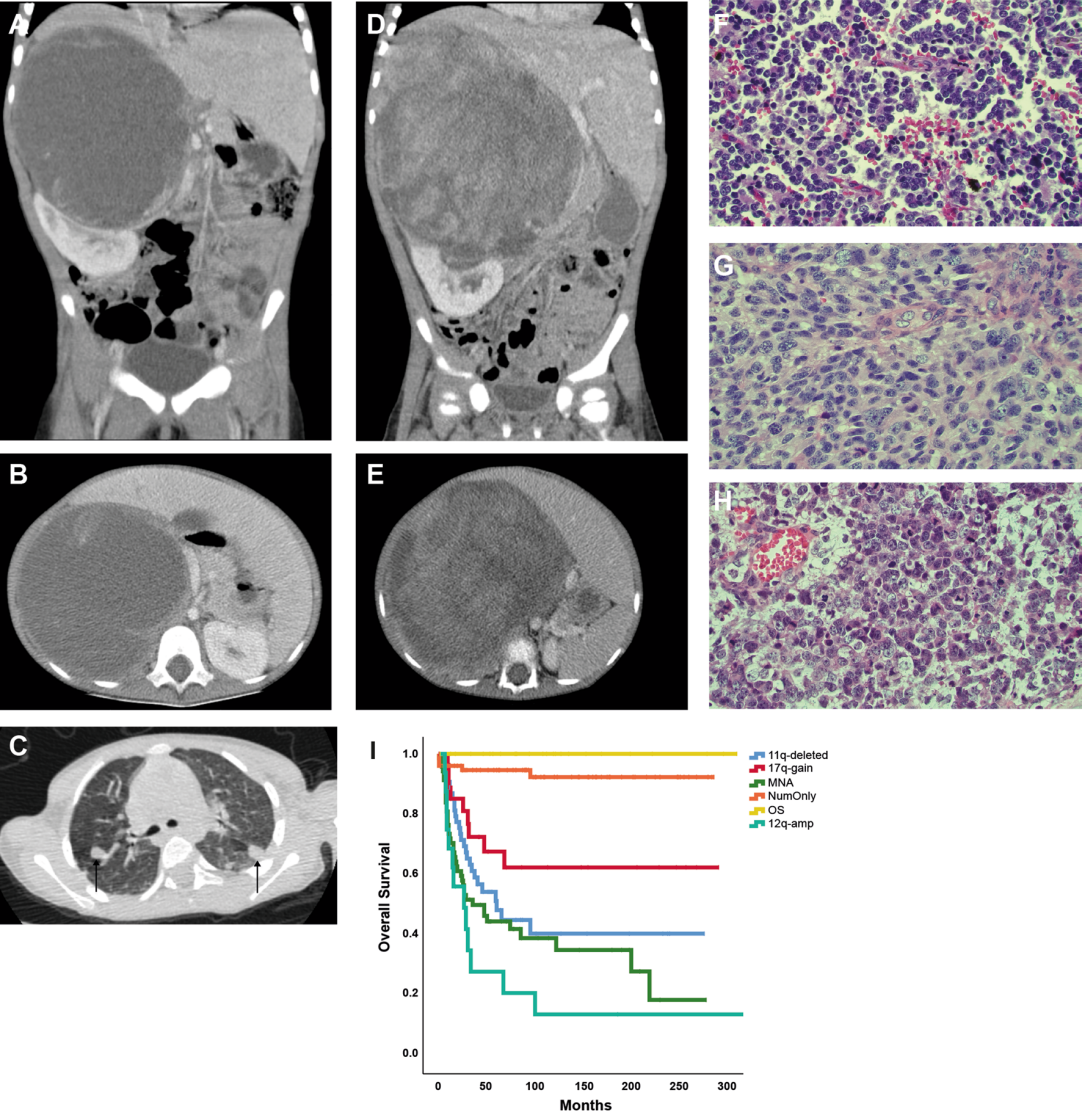
Clinical presentation of 12q-amplified tumors. (**A**,**B**) CT images of patient 45R1 showing a large primary NB tumor adjacent the upper part of the right kidney and (**C**) metastases of the lungs as indicated by arrows. (**D**,**E**) Corresponding images of a Wilms’ tumor with comparable growth pattern in a child at the similar age. Hematoxylin–eosin staining showing: (**F**) conventional poorly differentiated neuroblastoma with uniform nuclei and incipient resetting, patient 17E2, (**G**) large cell neuroblastoma with partly spindling cells and atypical mitoses, patient 45R1 and (**H**) undifferentiated large and anaplastic cell neuroblastoma with coarse nuclear chromatin, prominent nucleoli and atypical mitoses, patient 38R3. (**I**) Kaplan–Meier overall survival analysis show upon poor outcome among NB patients with 12q amplification in comparison to NB patients of different genomic subgroups.

As shown in Table 1, out of 17 patient cases for which detailed clinical data were available, seven had localized disease and ten had metastatic spread at time of diagnosis. The 12q-amplified NBs were all of abdominal origin, of which six had a renal location, and not surprisingly initially aroused suspicion of Wilms’ tumor, exemplified by CT images of NB case 45R1 (Fig. 2A–C) and a Wilms’ tumor from a patient of similar age (Fig. 2D–E). A subset of NB tumor hallmarked by 12q-amplification showed atypical large cell morphology compared to other genomic subgroups of NB showing conventional type of nuclei (Fig. 2F–H).

Moreover, the metastatic pattern showed statistically significant difference from what is common for high-risk NB, with a very low incidence of bone marrow involvement (4/10 = 40%) in our cohort as compared to the HR-NBL1/SIOPEN study reporting bone marrow involvement in 76.7% of included cases (375/489)^33^ (p = 0.015, Fisher’s exact test). Instead, lung metastases were favored (6/10 = 60%), that is an uncommon feature reported by the international neuroblastoma risk group project to be present in 3.6% of all NB (100/2808)^34^ (p > 0.00001, Fisher´s exact test). Kaplan–Meier analysis of 270 NB cases show that patients with 12q-amplified NB has extraordinary poor outcome in comparison to patients with tumors of other genomic subgroups (i.e. MNA, 11q-deleted, numerical only, 17q-gained or other segmental, as described previously^7^) (Fig. 2H). Among the patients with 12q-amplification, two of six patients with localized disease show long-term survival (> 300 months) while nine out of ten with metastasized disease had fatal outcome.

### Mutational analysis

Whole-genome sequencing (WGS; *n* = 3) and whole exome sequencing (WES; *n* = 5) were performed in tumors with 12q amplification from eight patients. Somatic calling for the four tumors for which constitutional DNA was available for sequencing (WGS: *n* = 3, WES: *n* = 1) detected an average of 18 nonsynonymous mutations (range 7–29; Supplementary Table 3). For tumors sequenced without matched normal controls (WES, *n* = 4), we focused on rare nonsynonymous variants in 574 genes with established connections to cancer (Supplementary Table 1). Using this approach, an average of 11 variants remained (range 7–19) (Supplementary Table 3) among the “tumor only” cases. Among these variants, we did not detect any novel nonsynonymous SNVs within the amplified regions at chromosome 12, except a rare *INHBE* variant seen in one patient. No variants affecting *ALK*, *PHOX2B*, *NRAS*, *KRAS*, or *TP53* were identified in the investigated tumor or germline DNA. However, three rare SNVs in *ATRX* were detected among the eight 12-amp tumors analyzed by sequencing. Alterations in genes connected to telomere maintenance (i.e., *TERT* and *ATRX*) have recently been shown to be recurrent in non-*MYCN*-amplified NBs, which are also connected to adverse outcome^12^. The *ATRX* SNVs included a novel nonsense variant in exon 13 (NM_000489.5; c.3979G > T; p.E1327*) in case SpHM and a novel missense variant in exon 30 (NM_000489.5; c.6327T > G; p.S2147R) in case AuHH, which is predicted to be damaging by altering the protein function. Moreover, an uncommon hemizygous *ATRX* missense variant (rs200709847) was detected in 34R0 (Supplementary Table 3). Furthermore, a somatic missense mutation in case (59R9) was detected in *SIN3A*, a gene recently associated with the repression of *TERT* gene expression^35^.

In addition to sequence analysis, re-examination of array data showed intergenic *ATRX* deletion in one case (BG12), while segmental alterations in the proximity of the *TERT* locus were detected in three cases: 16R0, SpAI, and in the NGP cell line. The breakpoints for the segmental alterations in the two non-*MYCN*-amplified tumors were located approximately 100 kb proximal to the *TERT* transcriptional starting point, while the corresponding breakpoint in NGP was located 1 Mb from *TERT*.

**Figure 3:**
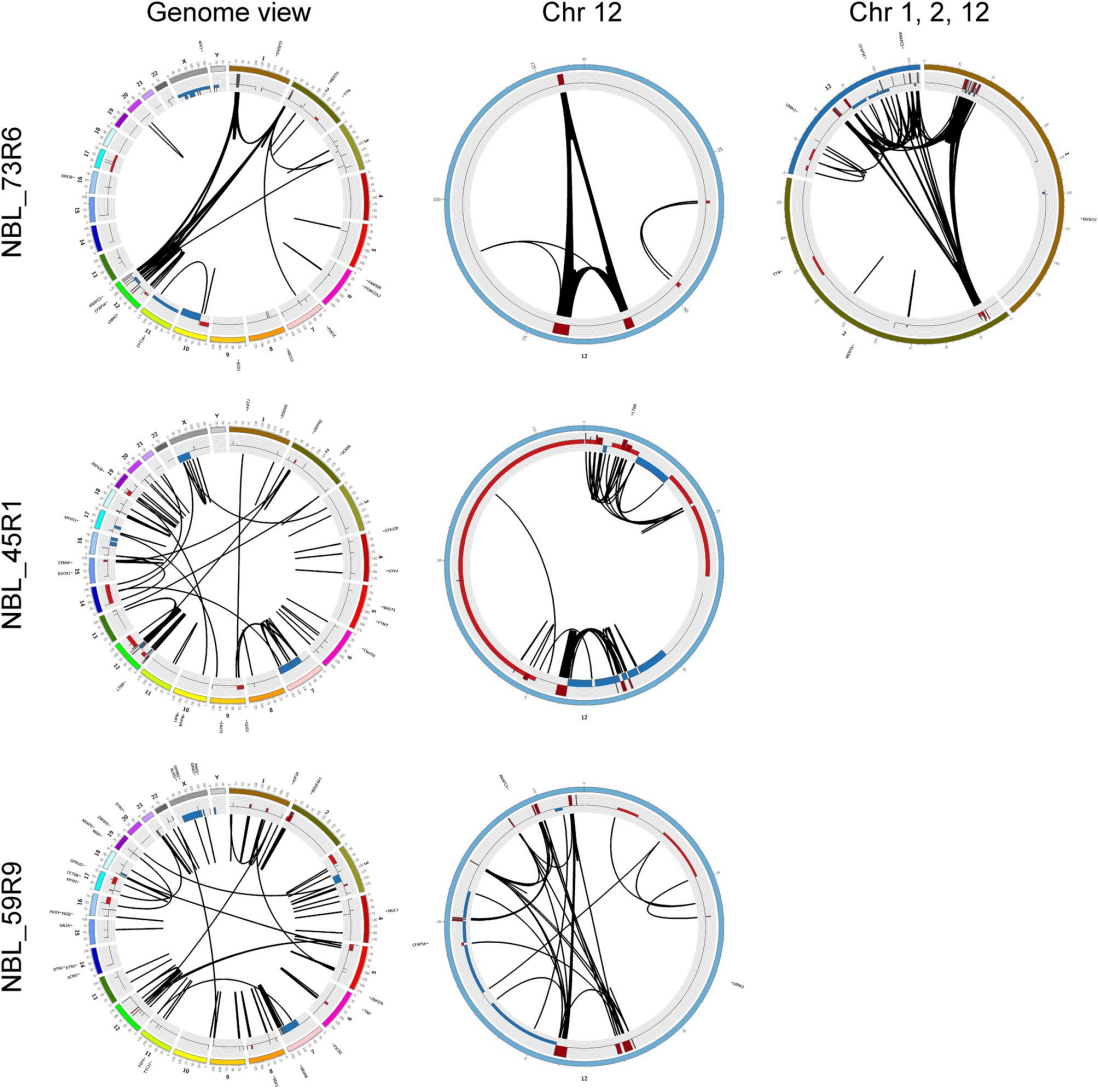
Mutational spectra and structural variants in 12q-amplified tumors. Circos plots showing structural variants, CNAs, and somatic SNVs. Copy number plots calculated based on the coverage ratio between tumor and corresponding normal tissue are shown on the inner circle, with gain of genomic material indicated in red and loss of genomic material indicated in blue. The lines within the inner circle indicate structural variants within and between chromosomes, while genes affected by somatic SNVs are shown outside the outer circle.

CNAs identified by WGS analysis of three primary tumors were in complete concordance with SNP microarray profiles (Fig. 1A and Supplementary Fig. 1), including amplified regions. WGS also showed extensive structural rearrangements between and within amplified regions (Fig. 3 and Supplementary Table 4).

### TP53 status in NB cell lines

Prior to treatment with Nutlin-3a, ribociclib, and abemaciclib in NB cell lines, different genomic aspects of the cell lines were characterized by SNP microarray analysis (Supplementary Fig. 2) together with mutational validation of previously reported *TP53* mutations^36–39^ by Sanger sequencing or by RT-PCR. The four cell lines (KELLY, SK-N-BE(2), SK-N-DZ, and SK-N-FI) displayed pathogenic homozygous missense mutations, rendering p53 inactive, whereas NGP has a *TP53* heterozygous mutation which allows a wild-type function of p53 (Supplementary Fig. 4A). RT-PCR verified the deletion of exons 10 and 11 of *TP53* that causes a protein truncation of the p53 C-terminus in SK-N-AS (Supplementary Fig. 4B). Relevant genetic alterations detected in the NB cell lines used in this study are summarized in Supplementary Fig. 4C.

### Treatment response to Nutlin-3a, ribociclib, and abemaciclib

**Figure 4:**
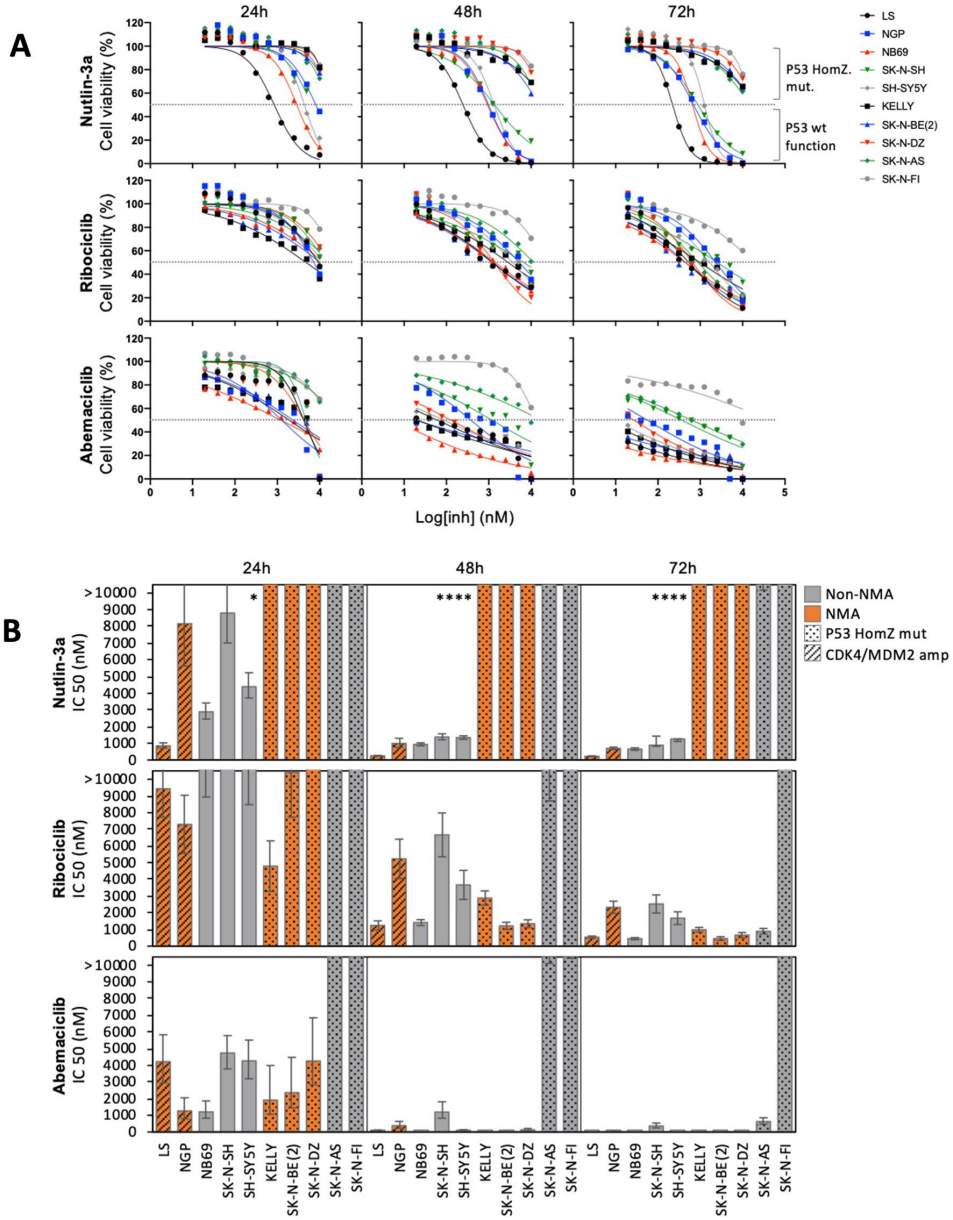
Neuroblastoma cell line response to MDM2i or CDK4/6i. (**A**) Normalized cell viability after treatment with Nutlin-3a (upper panels), ribociclib (middle panels), and abemaciclib (lower panels) for 24, 48, and 72 h. (**B**) IC50 values calculated after 24, 48, and 72 h of treatment. The bars represent the 95% CI for each IC50 value with statistical significance indicated as follows: *p < 0.01, **p < 0.001, *** *p* < 0.0001, and ****p < 0.00001.

To study whether cells with the co-amplification of *MDM2* and *CDK4* can benefit from inhibition directed toward these specific targets, we performed viability studies using MDM2i and CDK4/6i in NB cell lines. For inhibition of MDM2, Nutlin-3a (a selective antagonist of p53-MDM2 binding) was used, while two highly selective inhibitors, ribociclib and abemaciclib, were used for CDK4 inhibition. MDM2 inhibition showed that five out of ten cell lines were sensitive to Nutlin-3a treatment with 50% cell viability reduction already present after 24 h, while the remaining cell lines were resistant to Nutlin-3a even after 72 h of treatment (Fig. 4A, upper panels). The drug concentrations that cause a 50% reduction in cell viability (IC50 values) already produce a significant difference in the case of Nutlin-3a treatment between p53-mutated and p53^wild-type^ (p53^wt^) cell lines after 24 h of treatment (Fig. 4B, upper panel).

The response of Nutlin-3a was highly dependent on *TP53* mutational status, with treatment-resistant cell lines having homozygous mutated/deleted *TP53* while sensitive cell lines had at least one wild-type copy of *TP53*, such as NGP, which has a heterozygous *TP53* mutation (Supplementary Fig. 4). Regarding CDK4/6i, nine out of ten cell lines displayed a significant response to CDK4/6 inhibition with ribociclib (Fig. 4A, middle panels) or abemaciclib (Fig. 4A, lower panels). Of all investigated cell lines, only SK-N-FI displayed resistance to both ribociclib and abemaciclib treatment (Fig. 4A). No significant difference in IC50 values and response was detected between two *CDK4/MDM2*-amplified cell lines and non-amplified cell lines after treating the cells with CDK4i (Fig. 4A,B, middle and lower panels). *CDK4*/*MDM2* amplification did not have any significant impact on treatment response in comparison with other TP53^wt^ cell lines after MDM2i treatment (Fig. 4A,B, upper panels). No obvious response pattern in relation to the *MYCN* amplification and inhibition of CDK4 or MDM2 could be observed. For the detailed response of each treatment and cell line, see Supplementary Fig. 5, while details related to IC50 values for each cell line, treatment, and time point can be found in Supplementary Fig. 6.

### Synergy effect of combinatorial CDK4/6 and MDM2 inhibition

**Figure 5:**
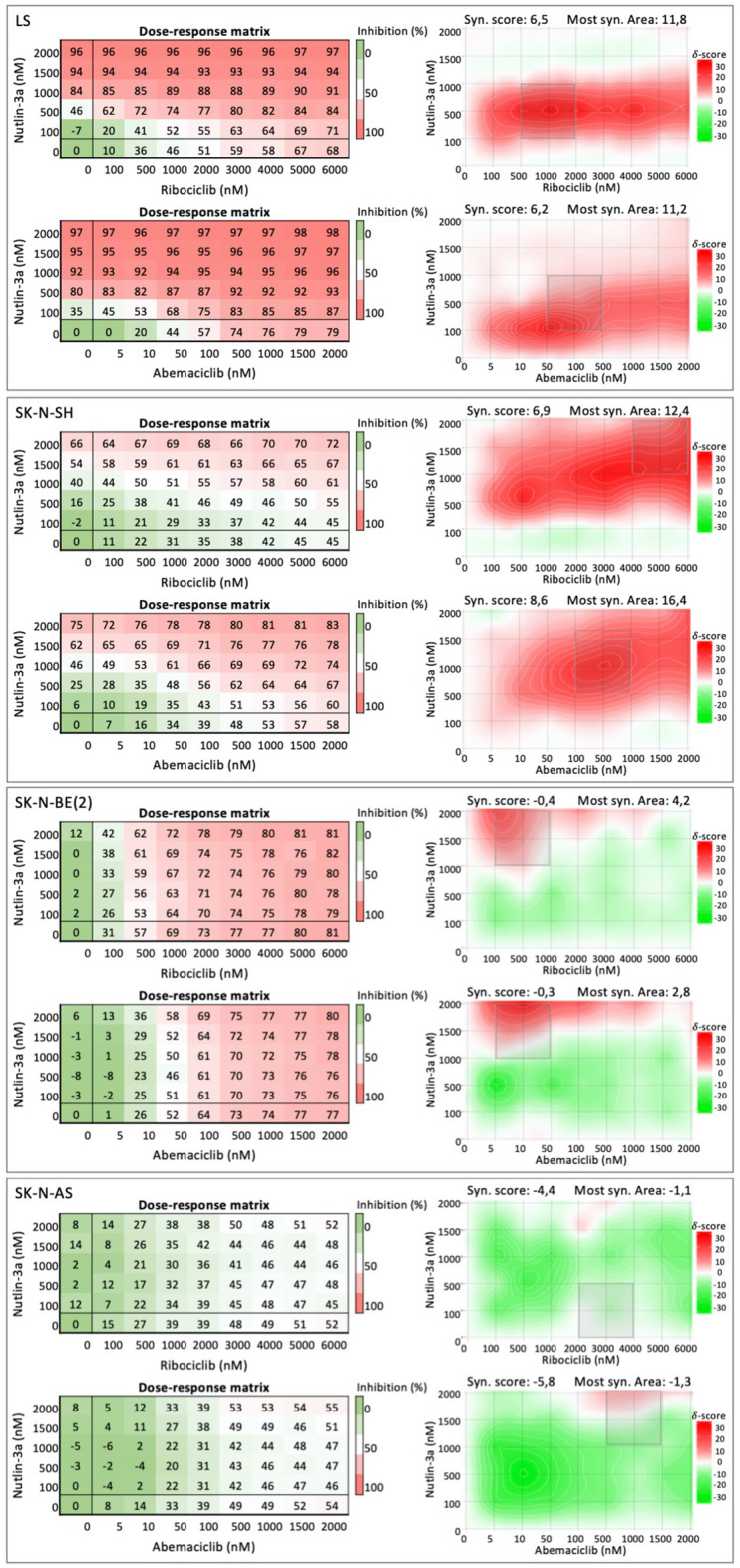
Inhibition dose–response matrix and synergy score heatmaps for representative neuroblastoma cell lines. Dose–response matrix and synergy score heatmaps generated by SynergyFinder for combinational treatment with Nutlin-3a and ribociclib and with Nutlin-3a and abemaciclib for the cell lines LS (*CDK4/MDM2*-amp, p53^wt^, MNA), SK-N-SH (p53^wt^, MNA), SK-N-BE (p53^mut^, MNA), and SK-N-AS (p53^mut^). Dose–response matrix and synergy score heatmaps for all used cell lines can be found in Supplementary Fig. 5.

Combination treatment with MDM2i and CDK4/6i did not result in a synergetic effect, furthermore the synergistic pattern was not conclusive, for this reason, the overall synergy score was used in order to interpret the results. The combination treatment induced a slight reduction in proliferation compared with single-agent treatment in the cell lines with p53^wt^, showing a positive score in the overall synergy score (Fig. 5, Table 3). In more detail, the p53^wt^ NB cell lines displayed a synergetic area greater than 10, meaning that the response is over 10% greater than expected at certain drug concentrations. Given the calculated overall synergy scores and the score for the most synergetic area, we can conclude that the combination of MDM2i + CDK4/6i has a positive additive effect in p53^wt^ NB cell lines. No significant difference in drug combination response was seen in LS and NGP, *MDM2*/*CDK4*-co-amplified cell lines, as compared with other p53^wt^ cell lines (Fig. 5, Table 3).

**Table 3:** Synergy scores after 72 h of combinational treatment.

Cell line	Drug combinations			
	Ribociclib + Nutlin-3a		Abemaciclib + Nutlin-3a	
	Overall synergy score	Most synergetic area	Overall synergy score	Most synergetic area
LS	6.5	11.8	6.2	11.2
NGP	8.4	15.1	5.3	12.0
NB69	4.7	10.5	6.7	12.5
SK-N-SH	6.9	12.4	8.6	16.4
SH-SY-5Y	10.1	16.6	8.3	15.2
KELLY	− 0.9	3.3	− 0.4	4.2
SK-N-BE(2)	− 0.4	4.2	− 0.3	2.8
SK-N-DZ	− 0.3	4.6	− 1.5	1.3
SK-N-AS	− 4.4	− 1.1	− 5.8	− 1.3
SK-N-FI	− 7.5	− 1.2	− 7.2	− 3.3

For the p53^mutated^ (p53^mut^) cell lines, the synergy score of three cell lines (KELLY, SK-N-BE(2), and SK-N-DZ) is close to 0, meaning that there is no difference between the single and combination treatments. These cell lines experienced a slight additive effect at specific concentrations of the combined drugs. The remaining two p53^mut^ cell lines (SK-N-AS and SK-N-FI) presented an antagonistic additive overview synergy score having a negative score in the most synergetic area. SK-N-AS displayed a later response to CDK4/6i with abemaciclib than did the rest of the cell lines, while SK-N-FI showed no significant response to CDK4/6i or MDM2i after 72 h of treatment (Fig. 4A,B).

### Protein levels of the CDK4/6 and MDM2 pathways

**Figure 6:**
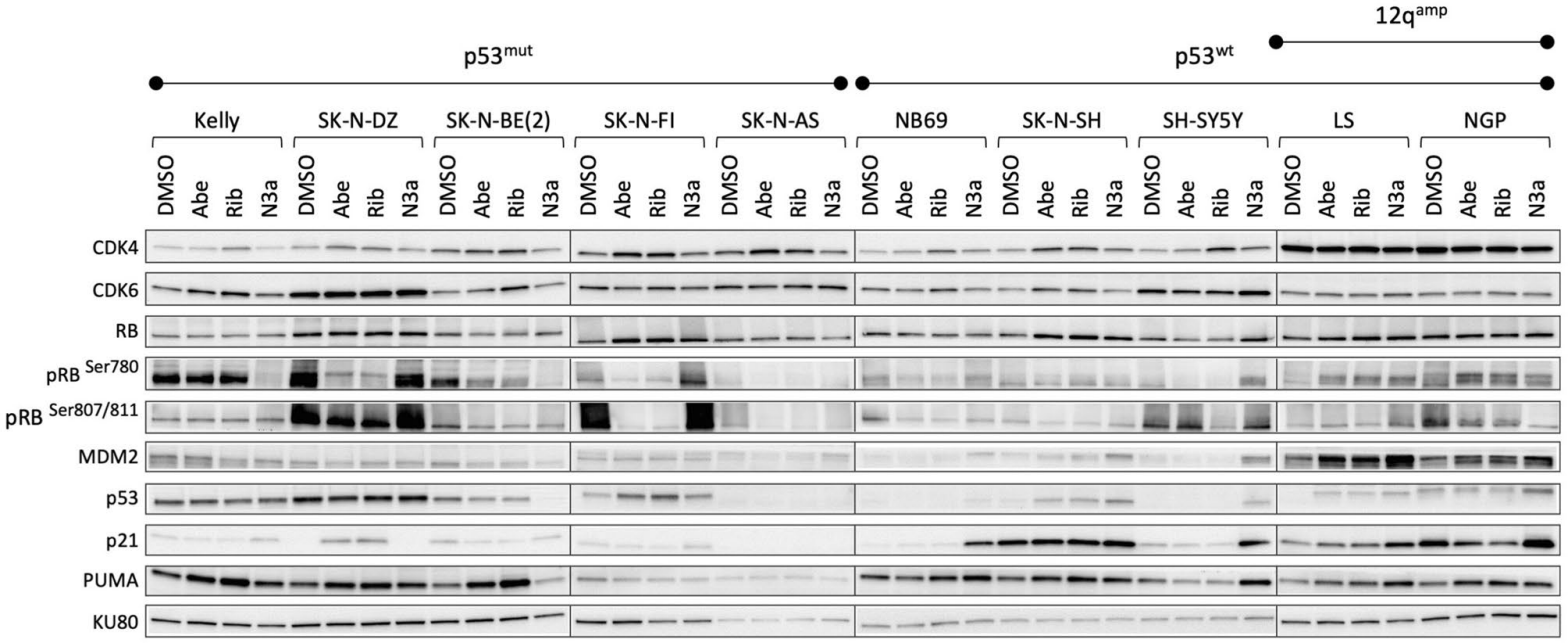
Effect on proteins of the CDK4 and MDM2 pathway after CDK4/6 or MDM2 inhibition. The cells were treated with abemaciclib (Abe), ribociclib (Rib), and Nutlin-3a (N3a) at IC50 values for 72 h before protein extraction. This figure is a representation of the protein expression using different blots. The blots are delineated to clarify that belong to different gels or to different parts of the same gel. Each blot was normalized using KU80 as control. Exposure time for each protein analysis was the same for all cell lines.

Downstream proteins of the CDK4/6 and MDM2 pathways were analyzed to verify the effect of the inhibitors used for each cell line at its respective IC50 value. CDK4/6 inhibition by both abemaciclib and ribociclib led to a marked decrease in RB phosphorylation levels at Ser780 in most used cell lines, except for the *CDK4* and *MDM2* co-amplified cell lines (LS and NGP), which have a higher expression than that of the experimental control. Regarding the phosphorylation of RB at Ser807/811, a similar pattern was found, except for KELLY, a cell line that had no changes in the expression at all. In the case of NGP, a decrease in pRB Ser807/811 has been observed (Fig. 6 and Supplementary Fig. 8). CDK4/6i also evoked increased protein levels of CDK4 (in all cell lines) and CDK6 (in all cell lines except for SH-SY5Y, LS, and NGP). Interestingly, CDK4/6i also led to increased levels of PUMA, a downstream target of p53, in four out of the five p53^mut^ cell lines (KELLY, SK-N-DZ, SK-N-BE(2), and SK-N-AS).

MDM2 inhibition with Nutlin-3a led to increased levels of p21 and MDM2 in all p53^wt^ cell lines. MDM2i also produced an overexpression of PUMA in all p53^wt^ cell lines as well as in KELLY and SK-N-DZ. We also detected that, after Nutlin-3a treatment, all cell lines (with the exception of SK-N-BE(2)) experienced an increase in p53 (Fig. 6).

## Discussion

In this study we investigated the amplification of two common regions at 12q13.3–14.1 and 12q15 detected in a subgroup of NB tumors. These two regions at chromosome 12 presented co-amplification in 15 out of 19 analyzed NB samples, and gene expression analysis showed that most of the genes in the amplicons were over-expressed compared with other NB tumors (Table 2). In other tumor types displaying the co-amplification of 12q13.3–14.1 and 12q15, *CDK4* and *MDM2* are often reported as possible targets for the respective regions^25,28–30^, and in our cohort of 12q-amplified cases, amplification of *CDK4* was seen in 18 out of 19 samples, while amplification of *MDM2* was seen in 16 out of 19 samples (Fig. 1 and Supplementary Fig. 1). Fibroblast growth factor receptor substrate 2 (*FRS2*), also implicated as an oncogenic driver in the 12q15 locus, was amplified in 14 out of the 19 cases with 12q amplification (Fig. 1D). The emergence of gene amplification is still not fully understood but likely involves a multistep process starting with an initial catastrophic event causing DNA breaks followed by retention of cells for which the alteration provide a selective advantage relative its environment, for review see Matsui et al.^40^. Our previous analyses of NB show that the *MYCN* amplicon occasionally is accompanied by several additional amplification peaks spread over chromosome region 2p^41^. The amplified regions are rarely larger that 3–4 Mb and suggests that carrying very large amplified segment provides (e.g. > 10 Mb) is a proliferative disadvantage for the cell. This could be due the risk of including less advantageous genes present in the larger amplicon or the replicative burden of carrying large amplification. Among the investigated cases with 12q-amplification, no one displayed one single, large continuous amplified segment including *CDK4*, *MDM2* and *FRS2*. Instead, the tumors most often presented two distinct amplification peaks; one centered at *CDK4* and another centered at *MDM2* (and *FRS2*). The specificity of this complex, yet highly specific amplification of these genes, suggests a very strong combined tumorigenic driver effect based on cooperative function.

Interestingly, among the 12q-amplified NB samples, five also had focal gain or amplification at 11q involving *CCND1* encoding cyclin D1*.* Cyclin D1 interacts with CDK4/6 and has a crucial role in G1/S transition by regulating RB phosphorylation^42^. Hence, *CCND1* gain could limit the biological consequences of saturation that might occur with endogenous cyclin D expression levels.

Aberrant telomere maintenance in NB is promoted by alterations of *ATRX* or *TERT* or through *MYCN*-driven *TERT* re-expression and is linked to poor prognosis in NB^12,43^. Among the 19 cases in our cohort, segmental variants associated with the *ATRX* gene were found in two patient samples, while segmental rearrangements in the proximity of the *TERT* loci were detected in three cases. Among the eight patient samples investigated by WES/WGS, three had rare SNVs in *ATRX*, including a truncated mutation (Supplementary Table 3). Apart from *ATRX*, few somatic SNVs with obvious relevance for cancer were detected by WES/WGS with an overall low mutational load, which is concordant with previous studies^4–6,44^. The sequenced tumors lacked pathogenic variants in *ALK*, *PHOX2B*, *TP53*, or genes of the RAS-MAPK signaling pathway, which have previously been connected to NB, as well as variants in *WT1*, *TRIM28*, *FBXW7*, *NYNRIN*, or *KDM3B*, genes previously linked to Wilms’ tumor^45^.

The adverse outcome seen for most patients with 12q-amplified tumors (Fig. 2I) indicates that new therapies are needed to improve treatment of this group of NB patients. Both *MDM2* and *CDK4* have been identified as prioritized targets in NB^46^ in ongoing pediatric trials of the CDK4/6 inhibitors abemaciclib (LY2835219) and palbociclib (PD0332991) (ClinicalTrials.gov: NCT02255461, NCT02644460), while a trial of ribociclib (LEE011) has recently been completed^47^. MDM2 targeting agents such as HDM201 (Novartis) are being evaluated in NB (ClinicalTrials.gov: NCT02780128), while RG7112 (Hoffman-La Roche) is in trial in adult solid cancer (ClinicalTrials.gov: NCT00559533). It has been suggested that combinatorial therapies may increase the efficacy in patients with dysregulation of both *MDM2* and *CDK4.* Synergy between CDK4/6 and MDM2 inhibitors has been observed in preclinical studies in liposarcoma^48^, melanoma,^49^ and breast cancer^50^, although contradictory results have been reported regarding the synergistic effects of a combined MDM2 and CDK4 targeting strategy in sarcoma^48,51^.

CDK4 and/or MDM2 inhibition, either as single-drug or combined treatment, showed that most investigated cell lines were sensitive to CDK4i, although SK-N-F1 displayed resistance to both ribociclib and abemaciclib (Fig. 4A,B, middle and lower panels), possibly due to high expression of multidrug resistance protein 1 (MDR1). Sensitivity to MDM2 inhibition with Nutlin-3a was only seen in p53^wt^ cell lines (Fig. 4A,B, upper panels). The two cell lines with co-amplification of *MDM2* and *CDK4* displayed sensitivity to both MDM2i and CDK4/6i at levels like those of other p53^wt^ cell lines (Table 3), indicating that directed therapy could be beneficial, despite the amplification and overexpression of the respective target genes. The combination of MDM2i and CDK4i did not present synergetic effects concordant with a recent study of combined MDM2/CDK4i in NB^52^. According to the overall synergy score, a positive additive effect was found in p53^wt^ cell lines, including the MDM2/CDK4-amplified cell lines, even though non synergetic effect was detected. No positive or even antagonistic effect was seen when combining CDK6i and MDM2i in p53^mut^ cell lines due to lack of response to Nutlin-3a in these cells (Table 3). However, as less than 2% of NB patients have tumors with p53 mutations at the time of diagnosis^53,54^, this indicates that a large group of NB patients may benefit from MDM2i, and possibly in combination with CDK4/6i in some specific cases. However, the broad sensitivity of MDM2i and CDK4i in investigated cell lines show that *MDM2*- and *CDK4*-amplification per se are not reliable biomarkers for response or non-response. Furthermore, with the rapid development of novel small molecule inhibitors or other therapies follows exponential increase of possible drug combinations. This requires additional pre-clinical studies using organoids, PDXs or other model systems in order to provide a strong biological rational for appropriate drug combinations before advancement to human subjects. If proceeding, and given NB being a rare neoplasm, a tumor-agnostic approach would likely be more feasible to establish critical safety and efficacy factors of dosing, timing and, sequencing of regimes for combination treatment.

Clinically, the degree of malignant behavior varied between tumors with 12q amplification, ranging from a localized, triploid tumor with a favorable outcome after surgical treatment only, to primary metastatic disease with a rapid demise. Interestingly, atypical clinical features in some of those tumors causing fatal progression included; non-involvement of bone or bone marrow (the major site of metastatic disease in NB), intra renal primary site and, presence of an inferior cava tumor thrombus and lung metastasis, suggesting an advanced Wilms’ tumor (WT). The deceptive morphology initially also favored the idea of this tumor. The amplifications in the mentioned regions might promote clinical features related to metastatic homing and tumor invasion that mimic WT. The co-amplification of *CDK4/MDM2* has also been noted to change the location and clinical features of rare cases of inflammatory myofibroblastic tumors, causing them to be mistaken for gastric cancer with local invasion of the spleen and diaphragm^55^. Predilection for lung metastasis have been confirmed in several malignancies such as melanoma, sarcoma, kidney- and breast cancer, in which the preferential site for metastasizing also is subtype dependent^56^**.** Examination of gene signatures in breast cancer has shown that whereas the signature for bone metastasis was mainly differentiated by cell surface- and secretory proteins^57^, the signature for lung metastasis correlated with markers for poor prognosis, were less specific for environment and instead promoted aggressive growth and invasiveness. If corresponding gene signature pattern also is present in NB is to our knowledge unknown. NB has also been reported to display a remarkable plasticity relating to the neoplastic origin of NB^58–63^ that also allows transdifferentiation, with switching between adrenergic- and mesenchymal state. If 12q-amplification has effect on NB plasticity or cell state and whether this in turn has effect on infiltration and metastatic homing is not fully investigated. Ultimately, additional studies of disseminating NB cells, their capability to passage organ specific barriers and collaboration with the microenvironment are required to elucidate the specific molecular mechanisms triggered by 12q-amplification that change invasive growth and preferential site for metastatic colonization in NB.

In summary, we present a subgroup of NB characterized by amplification at chromosome 12q with subsequent overexpression of most genes within the amplified regions. Atypical features were observed associated with this subgroup that might correlate to a cell cycle deregulation phenotype given the persistent amplification of *CDK4*, which is further potentiated by *MDM2* as well as *FRS2* amplification in some cases. This study provides new information about the possibility of using MDM2i and CDK4/6i drug combination in p53^wt^ NBs, including tumors with *MDM2* and *CDK4* amplification, although the effect of MDM2 inhibition is highly dependent on the presence of functional p53 activity.

## Materials and methods

### Sample collection, patients, and ethical statement

All experiments were performed in accordance with the approved guidelines and regulations. Tumor tissue was collected after written or verbal informed consent was obtained from parents/guardians according to ethical permits approved by the local ethics committees of Karolinska Institute and Karolinska University Hospital, the Experimental Research Ethics Committee of the University of Valencia/INCLIVA and the Spanish Society of Pediatric Hematology and Oncology, the University of Vienna, Wroclaw Medical University, and Oslo University Hospital. Genomic DNA was extracted from fresh frozen tumor or blood using the DNeasy Blood & Tissue kit (Qiagen, Hilden, Germany) according to the manufacturer’s protocol. The patients’ clinical and genetic backgrounds are described in Table 1.

### Morphology

The handling of specimen followed recommendations in standard protocol. Fresh material was immediately taken for biologic research and the fixed tumors were treated according to conventional pathology practice to diagnose neuroblastic tumors. Classification followed the International neuroblastoma Pathology Classification (INPC)^64^.

All tumor samples in the present paper had unfavorable histology; undifferentiated, poorly differentiated or anaplastic/large cell.

### SNP microarray analysis

The Swedish samples (*n* = 436) were screened using Affymetrix SNP microarrays (Thermo Fisher Scientific, Waltham, MA) as described earlier^7,44^. In addition, 12 NB cell lines were also screened using SNP microarray analysis: IMR32, KELLY, NB69, SH-SY5Y, SK-N-AS, SK-N-BE(2), SK-N-DZ, SK-N-FI, SK-N-SH (ECACC, HPA Culture Collections, Salisbury, UK), SH-EP (ATCC, Manassas, VA), as well as the two 12q-amplified cell lines LS and NGP (kindly provided by Prof. Manfred Schwab, DKFZ Heidelberg, Germany). There were nine patients with tumors displaying high-grade 12q-amplification in the Swedish cohort. In addition to these samples, a selection of patients from Norway (*n* = 1), Spain (*n* = 3), Austria (*n* = 2), and Poland (*n* = 2) with tumors sharing a similar 12q-amplification pattern were also included (Supplementary Fig. 1). In total, 19 cases with 12q-amplification were included in the study (tumor material from 17 patients and two cell lines). For primary data analysis, GDAS software (Thermo Fisher Scientific) was used, while genomic profiles and amplicon boundaries were determined using either the Chromosome Analysis Suite (ChAS 3.3; Thermo Fisher Scientific) or the Copy Number Analyzer for Affymetrix GeneChip Mapping arrays (CNAG 3.0; Genome Laboratory, Tokyo, Japan; www.genome.umin.jp).

### Massive parallel sequencing

WGS was performed on from three patients with tumors displaying 12q amplification, as evaluated from SNP microarrays. DNA from tumor material and matched constitutional DNA were subjected to sequencing and bioinformatical handling as described previously^52^ Briefly, sequencing was performed on Illumina instrumentation (Illumina, San Diego, CA) at Clinical Genomics, SciLife Laboratories, Stockholm, Sweden for average read depths of 62, 32, and 74 for tumor samples 45R1, 59R9, and 73R6, respectively, and of 30, 36, and 36 for constitutional DNA, respectively. Mapping, realignment around indels, and variant calling were carried out using the Sentieon suite of bioinformatics tools (Sentieon Inc., Mountain View, CA) (Sentieon version v201808.03). The Canvas tool (version 1.38.0.1554)^53^ was used to call CNAs while structural variants were called using the Manta tool (version 1.1.1)^54^, with filtering based on germline variation, artifacts caused by problematic regions or, presence in the SweGen Variant Frequency dataset (https://swefreq.nbis.se/) or in our in-house set of normal controls. Patient specific origin of tumor-normal pairs was verified using a previously developed Python script that calculate the shared fraction from 400,000 SNPs^65^. In addition to WGS, whole exome sequencing (WES) was performed on NB tumors from five additional patients together with constitutional DNA from one of these (17E2) using the SureSelect All Exon V5 50 Mb kit (Agilent, Santa Clara, CA) according to the supplier’s protocol. Thereafter, pair-end sequencing was performed on a HiSeq2500 sequencing system (Illumina) targeting an average of 50–60× raw read depth (GATC, Konstanz, Germany; Otogenetics, Atlanta, GA). Read trimming and mapping to the human reference genome were done using the CLC Genomics workbench (Qiagen, Aahus, Denmark).

Only high-quality called SNVs with a minimum of 10% variant allele frequency and a total read coverage of ten were considered for further analysis and evaluation. All synonymous variants or variants in non-coding regions were excluded except those affecting canonical splice sites. For further filtration, only variants present in 574 genes with established connections to cancer (listed in Supplementary Table 1) were kept when analyzing variants from tumors sequenced without corresponding normal tissue. The remaining variants were assessed manually using the Integrative Genomics Viewer (IGV)^66^. All genomic positions are given according to the human reference genome hg19/GRCh37, which was also used for mapping both WGS and WES.

### Gene expression in the 12q amplicon regions

Total RNA was isolated from 36 tumors and two cell lines (LS and NGP) using ToTALLY RNA (Ambion/Thermo Fisher Scientific) prior cDNA synthesizing using the High Capacity cDNA Reverse Transcription Kit (Applied Biosystems, Foster City, CA). cDNA corresponding to 250–500 ng of total RNA was loaded in triplicate onto TLDA cards (Applied Biosystems) to determine the expression levels of 31 genes (Table 2) located within the two common amplicon regions at 12q, together with housekeeping genes *SDHA*, *HPRT1*, *GAPDH*, *GUSB*, and *UBC* as endogenous controls. Gene expression levels were calculated using the delta delta C(t) method^67^ with the geometrical mean of the five endogenous controls. Fold change was calculated comparing 12q-amplified tumors (*n* = 5) and 12q non-amplified tumors (*n* = 31). Mean expression levels were calculated and compared gene-by-gene by two-tailed independent-sample *t*-test.

Kaplan–Meier plots were generated for each assay with median expression value as cutoff. Overall survival (OS) was defined as the time between diagnosis and death of any cause at follow-up. The OS of the patients was compared with gene expression levels using a log rank test, with a *P*-value less than 0.05 considered significant. These data were then compared with two publicly available expression datasets in the R2 database (R2: Genomics Analysis and Visualization Platform, http://r2.amc.nl): the Versteeg (*n* = 88) data derived from GeneChip Human Genome U133 Plus 2.0 Array (Affymetrix) and the SEQC dataset (*n* = 498) derived from transcriptome sequencing.

### Cell culture, drug treatment, and viability assay

All NB cell lines were cultured under standard practice without antibiotics and routinely tested for mycoplasma. The cell line identity was verified by SNP microarray analysis (Supplementary Fig. 2) while *TP53* mutational status was confirmed by Sanger sequencing and RT-PCR.

Ten NB cell lines with different genetic backgrounds^68,69^ were used for MDM2 and CDK4 inhibition: KELLY, SK-N-BE(2), SK-N-DZ, SK-N-AS, SK-N-FI, SK-N-SH, SH-SY5Y, NB69, LS, and NGP. The cell lines were plated, 24 h before treatment, in 96-well plates at a density of 5000–10,000 cells/well depending on the doubling time of each cell line. The cells were treated with the selective MDM2 antagonist drug Nutlin-3a (Selleckchem, Houston, TX) and/or the selective CDK4/6 inhibitors abemaciclib (MedChemExpress, Monmouth Junction, NJ) or ribociclib (MedChemExpress, Monmouth Junction, NJ). Concentrations of the tested inhibitors ranged from 20 to 10,000 nM (two-fold dilutions); DMSO was used as a control in an amount corresponding to the levels present in the highest used drug concentrations. The cell viability was measured by means of fluorescence using Presto Blue SH cell viability reagent (Thermo Fisher Scientific). Cell proliferation was monitored for 24, 48, and 72 h. The experiments were carried out in six replicates at each concentration and repeated at least three times. The concentrations that caused 50% inhibition of cell growth (IC50) were calculated using GraphPad Prism, version 8.4.3 (GraphPad Software, San Diego, CA).

### Drug combination assay

To study a possible synergy effect on cell proliferation after dual MDM2 and CDK4/6 inhibition, cells were treated for 72 h with either (a) Nutlin-3a combined with ribociclib or (b) Nutlin-3a combined with abemaciclib. The concentration ranges were from 0 to 6000 nM for ribociclib and from 0 to 2000 nM for Nutlin-3a and abemaciclib. The viability was normalized as the percentage of inhibition prior to calculating the synergy score for drug combination responses, which were calculated based on the HSA reference model using SynergyFinder^70^. The derived synergy score data can be directly interpreted as the proportion of the cellular response associated with the drug interaction. As synergy scores near 0 have limited confidence, synergy scores between − 10 and 10 are considered to indicate additive interaction, with positive and negative score values denoting synergy and antagonism, respectively: synergy scores larger than 10 are considered to indicate synergetic interaction, whereas synergy scores below − 10 are considered antagonistic interaction^70^.

### Western blotting

Proteins from cells treated for 72 h with corresponding IC50 concentration for respective cell lines and control cells were extracted using RIPA lysis buffer supplemented with proteinase and phosphatase inhibitors. Protein amount was quantified using the Pierce BCA Protein assay kit (Thermo Fisher Scientific). The same amount of total protein sample (20 µg) was separated from each sample using SDS-PAGE and transferred onto 0.2-µm PVDF membrane. In order to optimize the number of proteins analyzed from a single membrane the blots were cut in multiple pieces according to size. Uncropped blot images are presented in Supplementary Fig. 9. The blots were probed overnight at 4 °C with the following antibodies: CDK4 (sc-56277), CDK6 (sc-7961), MDM2 (sc-965), p53 (sc-126), PUMA (sc-374223), and RB (sc-102) from Santa Cruz Biotechnology (Dallas, TX); p-RB^Ser807/811^ (#702097) from Thermo Fisher Scientific; and p-RB^Ser780^ (#9307), p21 (#2947), and KU80 (#2573) from Cell Signaling Technology (Danvers, MA). Protein levels were quantified using ImageJ software^60^ with normalization against KU80.

### Statistical analysis

The Kaplan–Meier estimates are based on data generated from 270 neuroblastoma cases with available clinical outcome and the analysis was performed using the IBM SPSS statistics for Windows, version 25 while the other statistical analyses were performed using GraphPad Prism, version 8.4.3 (GraphPad Software). The IC50 values and corresponding 95% confidence intervals (95% CIs) were calculated using dose–response sigmoidal curve with variable slope analysis (the log [inhibitor] vs. normalized response–variable slope). The difference in the IC50 values of two groups (p53^mut^ vs. p53^wt^) was analyzed using Student’s *t*-test. *P* values < 0.05 were considered statistically significant.

## Supplementary Information

Supplementary Figures.

Supplementary Tables.
