# Supplementary material for: Amplification of CDK4 and MDM2: a detailed study of a high-risk neuroblastoma subgroup
Source: Sci Rep. 2022 Jul 20;12:12420. doi: 10.1038/s41598-022-16455-1 (PMC9300649; doi:10.1038/s41598-022-16455-1)
Supplement: Supplementary file 1 — Supplementary Figures. [file 41598_2022_16455_MOESM1_ESM.pdf]

Supplemental figures

Amplification of *CDK4* and *MDM2*: A detailed study of a high-risk neuroblastoma subgroup

Angela Martinez-Monleon[1], Hanna Kryh Öberg[1], Jennie Gaarder[1], Ana P. Berbegall[2], Niloufar Javanmardi[1], Anna Djos[1], Marek Ussowicz[3], Sabine Taschner-Mandl[4], Inge M. Ambros[4], Ingrid Øra[5], Bengt Sandstedt[6], Klaus Beiske[7], Ruth Ladenstein[4], Rosa Noguera[2], Peter F. Ambros[4], Lena Gordon Murkes[8,] Gustaf Ljungman[9], Per Kogner[10], Susanne Fransson[1] and Tommy Martinsson[1]

[1]Department of Laboratory Medicine, University of Gothenburg, Gothenburg, Sweden.
[2]Department of Pathology, Faculty of Medicine and Dentistry, University of Valencia, Valencia, Spain.
[3]Department of Pediatric Bone Marrow Transplantation, Oncology and Hematology, Wroclaw Medical University, 50-556 Wroclaw, Poland.
[4]Children's Cancer Research Institute (CCRI), St. Anna Kinderkrebsforschung, Vienna, Austria.
[5]Department of Pediatric Oncology and Hematology, Clinical Sciences, Lund University, Lund, Sweden.
[6]Childhood Cancer Research Unit, Karolinska Institutet, Stockholm, Sweden.
[7]Institute of Clinical Medicine, University of Oslo, Oslo, Norway.
[8]Department of Pediatric Radiology, Astrid Lindgren Children´s hospital, Karolinska University Hospital, Stockholm, Sweden
[9]Department of Women's and Children's Health, Children's University Hospital, University of Uppsala, Uppsala, Sweden.
[10]Department of Women's and Children's Health, Karolinska Institutet, Stockholm, Sweden.

Supplemental Figure 1. Global genome profile view.

Supplemental Figure 2. Copy number profile for verification of cell line identity.

Supplemental Figure 3. Comparison of MDM2 gene expression and DNA copy number for neuroblastoma tumors of different subgroups.

Supplemental Figure 4. TP53 alterations and p53 associated pathways.

Supplemental Figure 5. Viability of neuroblastoma cell lines after inhibition of MDM2 or CDK4/6.

Supplemental Figure 6. IC50 values (nM) for each treatment.

Supplemental Figure 7. Inhibition Dose-response matrix and synergy score heatmaps of neuroblastoma cell lines.

Supplemental Figure 8. Protein quantification of NB cell lines after been treated with CDK4i and MDM2i.

Supplemental Figure 9. Western blot images of used membranes

Supplemental Figure 10. Uncropped gel images used for Supplemental Figure 4C

Sup. Fig 1

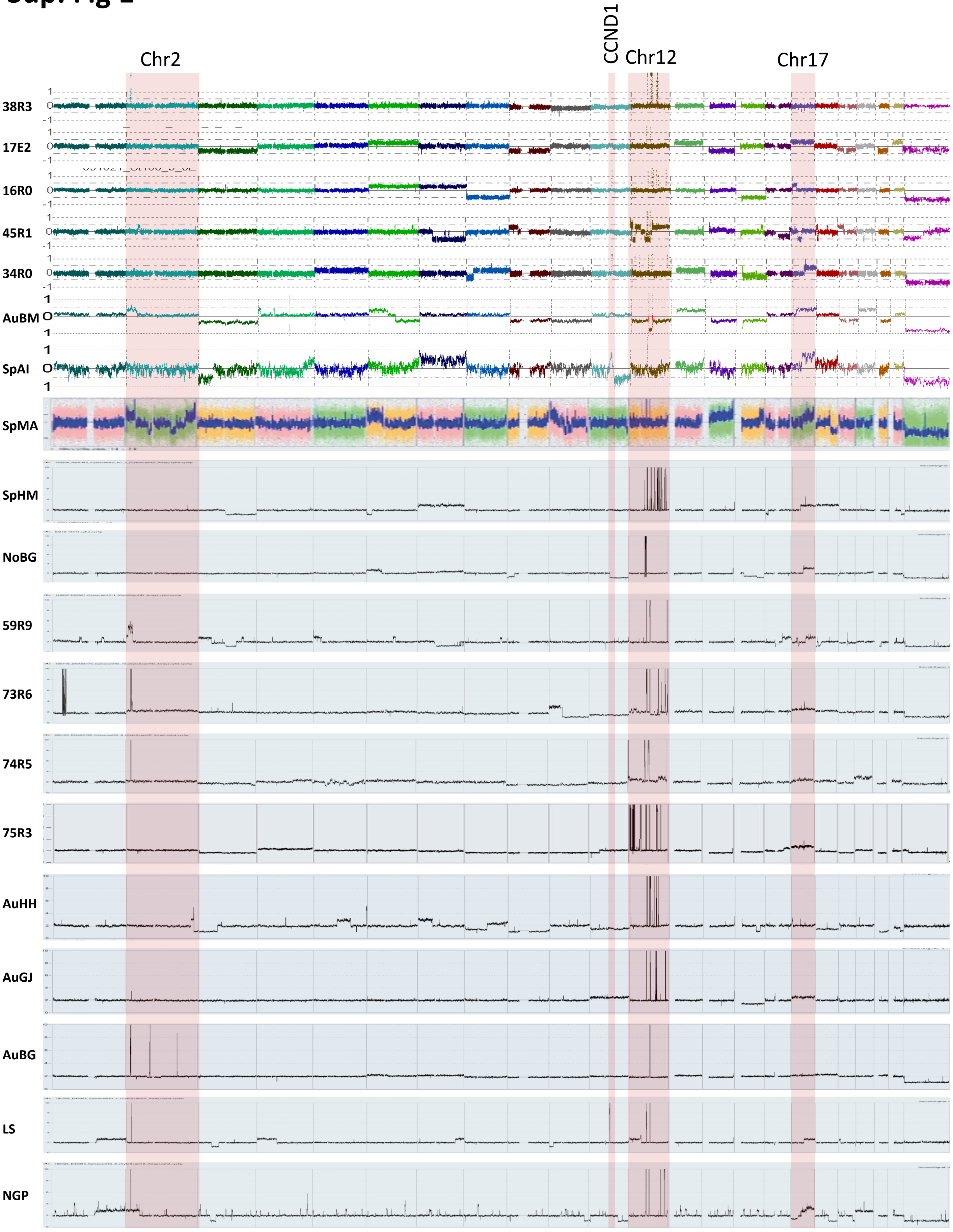

Supplemental Figure 1. Global genome profile view. Whole genome copy number profiles generated from SNP-microarray for the NB tumor samples included in this study.

Sup. Fig 2

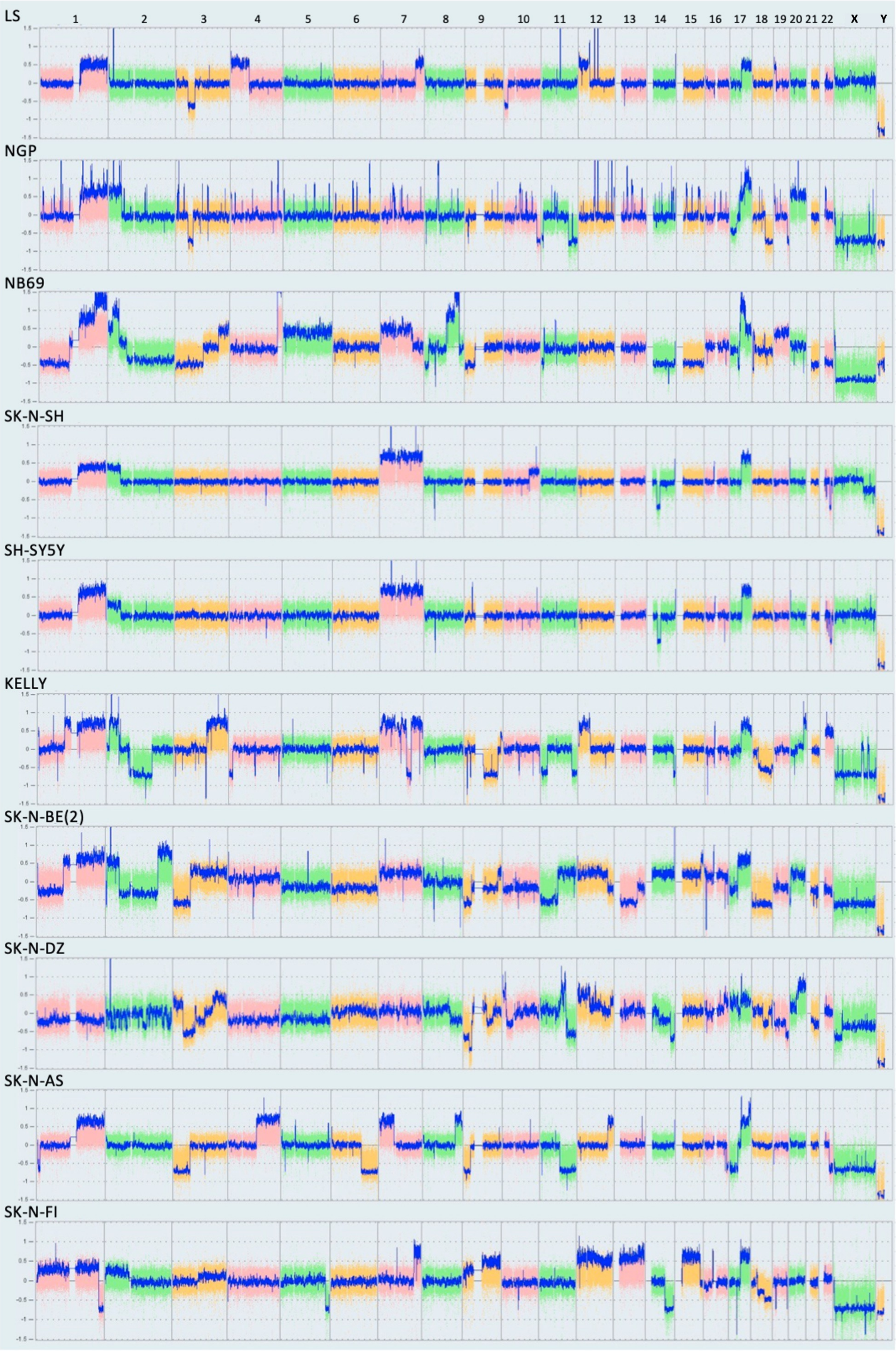

Supplemental Figure 2. Copy number profile for verification of cell line identity. Copy number profiles generated from SNP-microarray for verification of cell line identity and identification of larger genomic alterations in the ten NB cell lines used in this study.

Sup. Fig 3

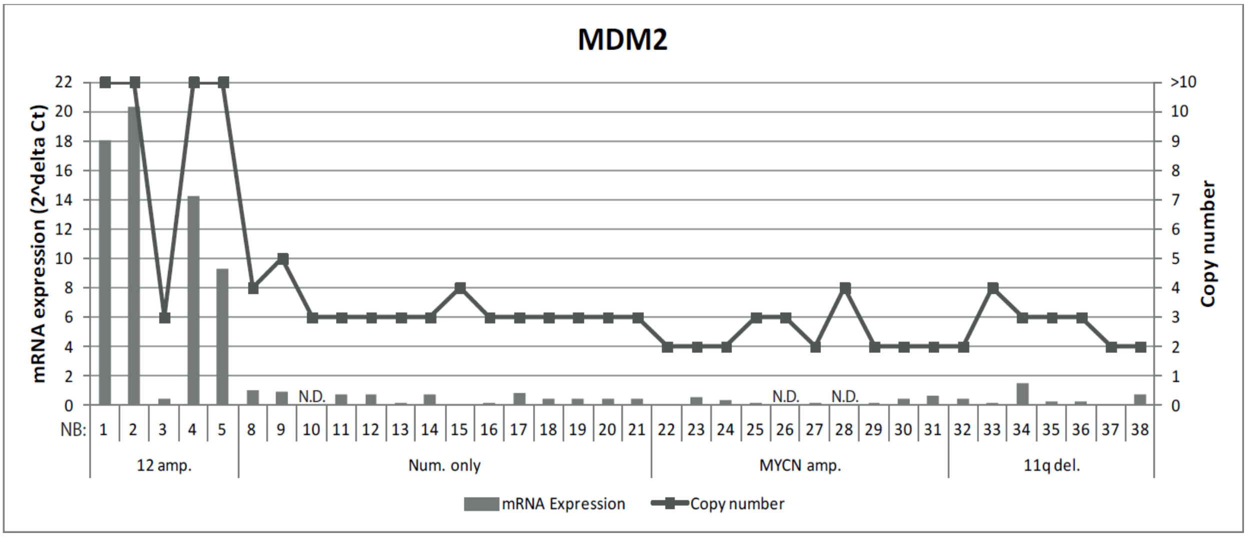

Supplemental Figure 3. Comparison of MDM2 gene expression and DNA copy number for neuroblastoma tumors of different subgroups. Note that the tumor NB3 (17E2) does not include *MDM2* in the 12q15 amplification peak at the position of *MDM2*. Similarly, NB3 (17E2) also lacks the high *MDM2* expression seen in the rest of this subgroup and instead resembles the NB tumors without any amplification at chromosome 12. Gene expression is presented as the mRNA expression of *MDM2* relative to a set of five endogenous control genes. Copy number data for the region covering the *MDM2* gene is inferred from the SNP-arrays. For high grade amplifications, where the exact copy number cannot be accurately measured due to a saturation of the signal, the copy number has been set to >10 copies.

Sup. Fig 4

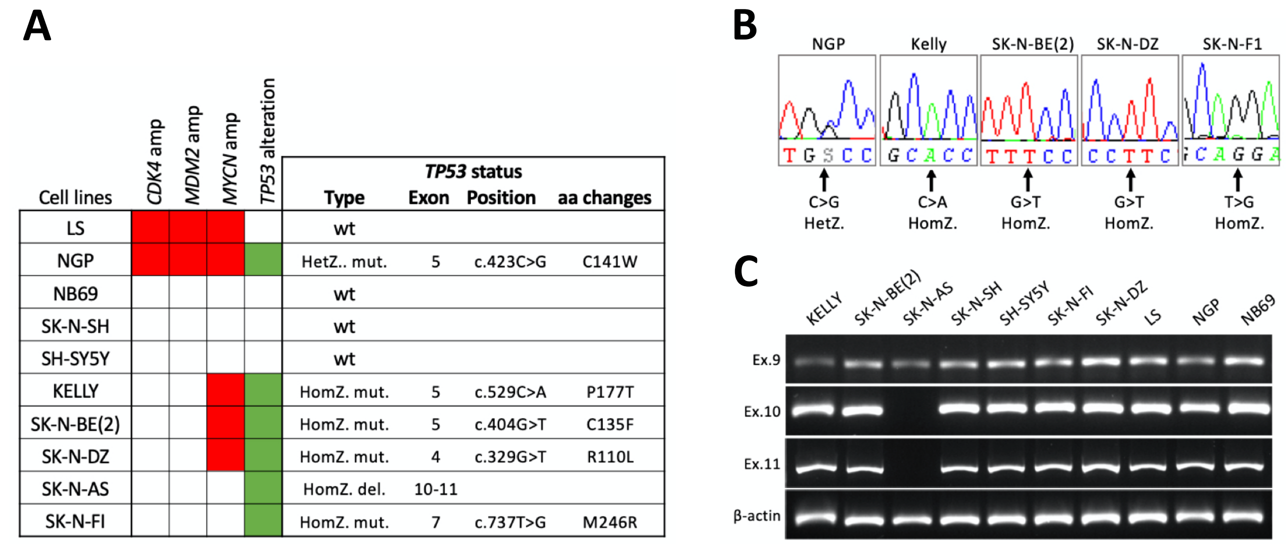

Cell lines	*CDK4* amp	*MDM2* amp	*MYCN* amp	*TP53* alteration	*TP53* status Type	Exon	Position	aa changes
LS					wt			
NGP					HetZ.. mut.	5	c.423C>G	C141W
NB69					wt			
SK-N-SH					wt			
SH-SY5Y					wt			
KELLY					HomZ. mut.	5	c.529C>A	P177T
SK-N-BE(2)					HomZ. mut.	5	c.404G>T	C135F
SK-N-DZ					HomZ. mut.	4	c.329G>T	R110L
SK-N-AS					HomZ. del.	10-11		
SK-N-FI					HomZ. mut.	7	c.737T>G	M246R

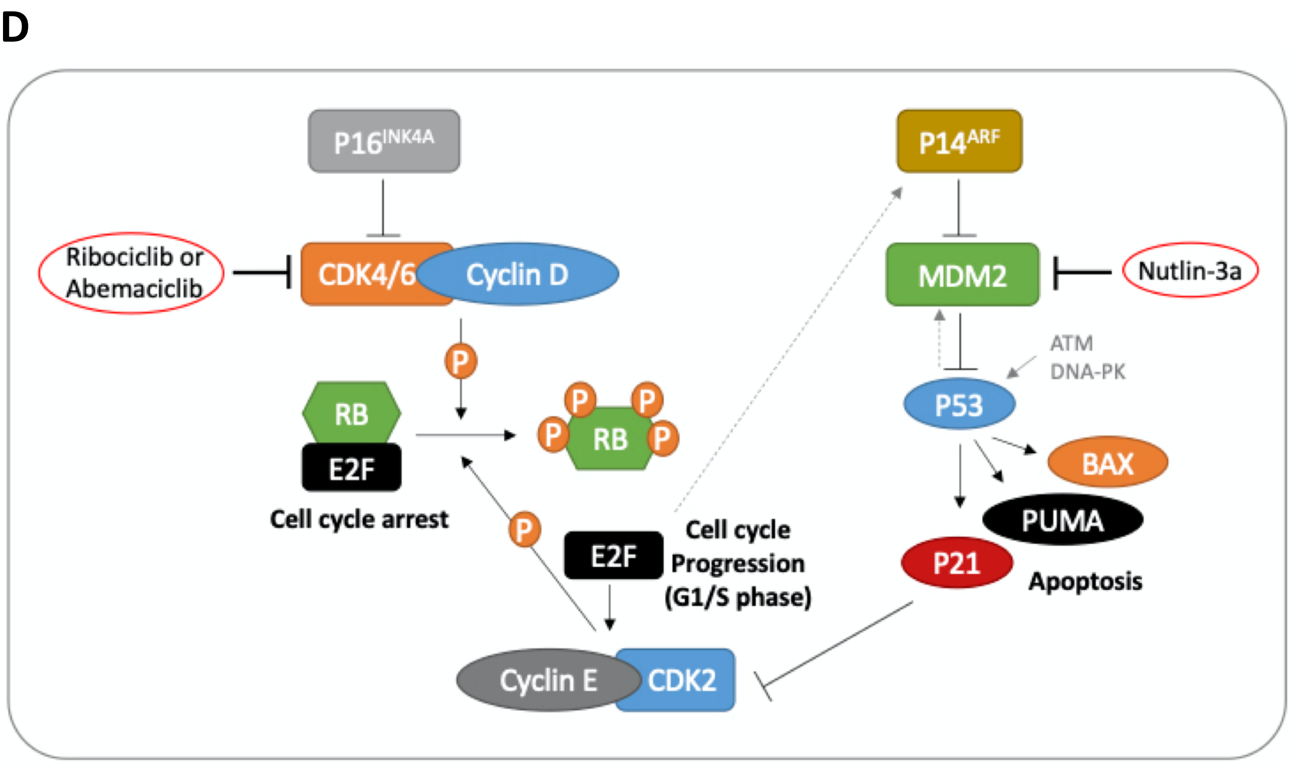

Supplemental Figure 4. TP53 alterations and p53 associated pathways. A) Summary of p53 mutational status and general characteristics in used cell lines. **B)** Electropherograms from Sanger sequencing showing homozygous missense mutations in four NB cell lines and a heterozygous missense mutation in one cell line (NGP). **C)** RT-PCR shows loss of exon 10 and 11 in SK-N-AS. Full gel images shown in Supplemental Figure 10. **D)** Overview of the CDK4/6/RB and MDM2/p53 pathways.

Sup. Fig 5

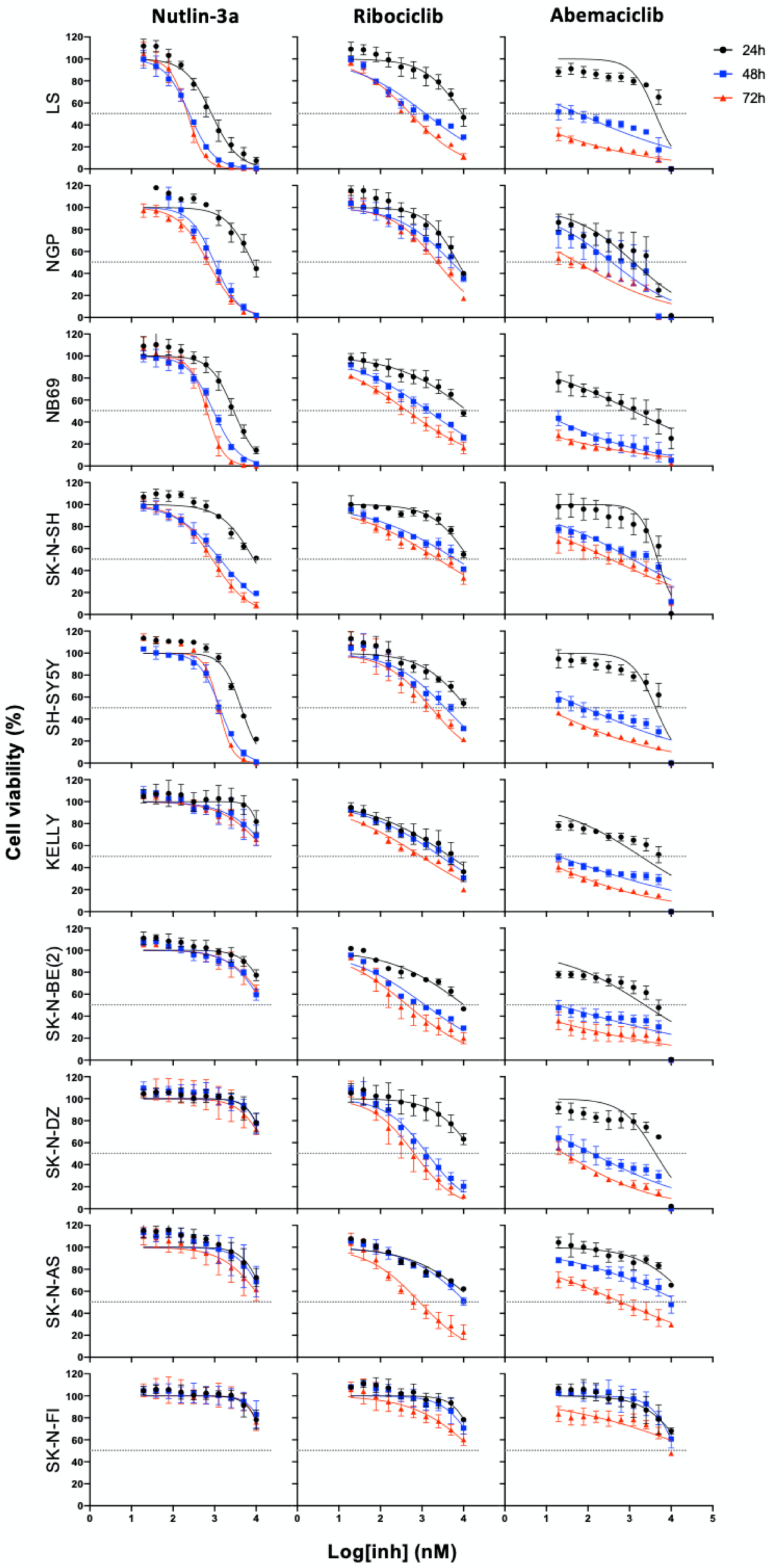

Supplemental Figure 5. Viability of neuroblastoma cell lines after inhibition of MDM2 or CDK4/6. Representation of the normalized cell viability after treatment with Nutlin-3a, Ribociclib and Abemaciclib during 24, 48 and 72h. The bars represent the standard deviation between replicates.

Sup. Fig 6

Cell line	Nutlin-3a					
	24h		48h		72h	
	IC50 (nM)	95% IC (nM)	IC50 (nM)	95% IC (nM)	IC50 (nM)	95% IC (nM)
LS	848,3	704,3 to 1026	247,3	227,1 to 269,3	222,4	201,3 to 245,8
NGP	8167	5639 to 14568	989,5	755,6 to 1302	694,3	625,5 to 770,1
NB69	2889	2452 to 3416	933,2	846,8 to 1028	652,3	585,3 to 726,0
SK-N-SH	8806	7009 to 11816	1376	1210 to 1568	886,4	816,9 to 961,9
SH-SY5Y	4391	3715 to 5227	1339	1240 to 1446	1258	1110 to 1428
KELLY	>10000	>10000	>10000	>10000	>10000	>10000
SK-N-BE(2)	>10000	>10000	>10000	>10000	>10000	>10000
SK-N-DZ	>10000	>10000	>10000	>10000	>10000	>10000
SK-N-AS	>10000	>10000	>10000	>10000	>10000	>10000
SK-N-FI	>10000	>10000	>10000	>10000	>10000	>10000

Cell line	Ribociclib					
	24h		48h		72h	
	IC50 (nM)	95% IC (nM)	IC50 (nM)	95% IC (nM)	IC50 (nM)	95% IC (nM)
LS	9427	7722 to 12163	1239	966,4 to 1615	534,3	470,6 to 607,4
NGP	7295	5553 to 10463	5231	4054 to 7090	2324	1959 to 2773
NB69	>10000	>10000	1411	1236 to 1618	443	380,9 to 515,2
SK-N-SH	>10000	>10000	6666	5350 to 8585	2526	1982 to 3312
SH-SY5Y	>10000	>10000	3666	2796 to 4990	1675	1293 to 2199
KELLY	4791	3274 to 7812	2881	2454 to 3427	979,2	837,4 to 1151
SK-N-BE(2)	>10000	>10000	1210	996,7 to 1482	448,3	333,0 to 607,2
SK-N-DZ	>10000	>10000	1341	1106 to 1636	654,8	496,1 to 870,5
SK-N-AS	>10000	>10000	>10000	>10000	887,6	720,2 to 1101
SK-N-FI	>10000	>10000	>10000	>10000	>10000	>10000

Cell line	Abemaciclib					
	24h		48h		72h	
	IC50 (nM)	95% IC (nM)	IC50 (nM)	95% IC (nM)	IC50 (nM)	95% IC (nM)
LS	4220	2943 to 5833	61,7	26,35 to 112,7	<20	<20
NGP	1274	800,9 to 2055	380,8	221,9 to 634,9	55,91	35,95 to 80,45
NB69	1212	827,0 to 1868	<20	<20	<20	<20
SK-N-SH	4737	3790 to 5783	1201	825,4 to 1815	363,1	241,3 to 537,6
SH-SY5Y	4268	3197 to 5516	87,37	48,87 to 138,6	<20	<20
KELLY	1918	1067 to 3992	20,14	6,837 to 40,74	<20	<20
SK-N-BE(2)	2363	1448 to 4477	<20	3,364 to 48,36	<20	<20
SK-N-DZ	4262	2830 to 6851	128,6	70,23 to 211,1	26,78	19,33 to 35,19
SK-N-AS	>10000	>10000	>10000	>10000	627,1	465,3 to 854,6
SK-N-FI	>10000	>10000	>10000	>10000	>10000	>10000

Supplemental Figure 6. IC50 values (nM) for each treatment. Concentrations of inhibitor needed for a 50% reduction of cell viability. IC50 values together with their 95% CI were calculated using GraphPad Prism 8.4.3.

Sup. Fig 7

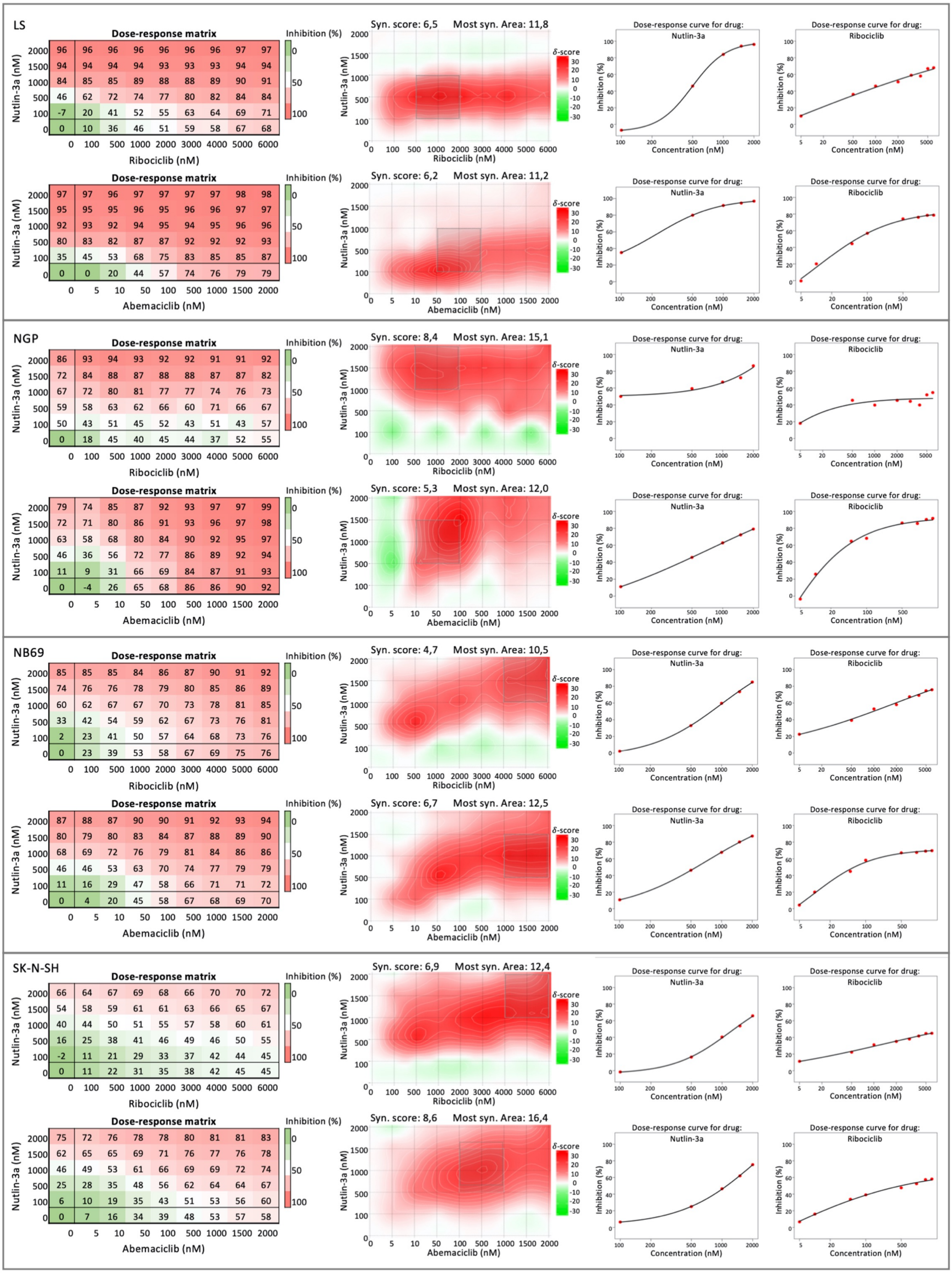

LS
Dose-response matrix
Inhibition (%)
Nutlin-3a (nM)
2000 96 96 96 96 96 96 96 97 97
1500 94 94 94 94 93 93 93 94 94
1000 84 85 85 89 88 88 89 90 91
500 46 62 72 74 77 80 82 84 84
100 -7 20 41 52 55 63 64 69 71
0 0 10 36 46 51 59 58 67 68
0 100 500 1000 2000 3000 4000 5000 6000
Ribociclib (nM)
Syn. score: 6,5 Most syn. Area: 11,8
δ-score
Dose-response curve for drug:
Nutlin-3a
Ribociclib
Inhibition (%)
Concentration (nM)
Dose-response matrix
2000 97 97 96 97 97 97 97 98 98
1500 95 95 95 96 95 96 96 97 97
1000 92 93 92 94 95 94 95 96 96
500 80 83 82 87 87 92 92 92 93
100 35 45 53 68 75 83 85 85 87
0 0 0 20 44 57 74 76 79 79
0 5 10 50 100 500 1000 1500 2000
Abemaciclib (nM)
Syn. score: 6,2 Most syn. Area: 11,2
NGP
Dose-response matrix
2000 86 93 94 93 92 92 91 91 92
1500 72 84 88 87 88 88 87 87 82
1000 67 72 80 81 77 77 74 76 73
500 59 58 63 62 66 60 71 66 67
100 50 43 51 45 52 43 51 43 57
0 0 18 45 40 45 44 37 52 55
0 100 500 1000 2000 3000 4000 5000 6000
Ribociclib (nM)
Syn. score: 8,4 Most syn. Area: 15,1
Dose-response matrix
2000 79 74 85 87 92 93 97 97 99
1500 72 71 80 86 91 93 96 97 98
1000 63 58 68 80 84 90 92 95 97
500 46 36 56 72 77 86 89 92 94
100 11 9 31 66 69 84 87 91 93
0 0 -4 26 65 68 86 86 90 92
0 5 10 50 100 500 1000 1500 2000
Abemaciclib (nM)
Syn. score: 5,3 Most syn. Area: 12,0
NB69
Dose-response matrix
2000 85 85 85 84 86 87 90 91 92
1500 74 76 76 78 79 80 85 86 89
1000 60 62 67 67 70 73 78 81 85
500 33 42 54 59 62 67 73 76 81
100 2 23 41 50 57 64 68 73 76
0 0 23 39 53 58 67 69 75 76
0 100 500 1000 2000 3000 4000 5000 6000
Ribociclib (nM)
Syn. score: 4,7 Most syn. Area: 10,5
Dose-response matrix
2000 87 88 87 90 90 91 92 93 94
1500 80 79 80 83 84 87 88 89 90
1000 68 69 72 76 79 81 84 86 86
500 46 46 53 63 70 74 77 79 79
100 11 16 29 47 58 66 71 71 72
0 0 4 20 45 58 67 68 69 70
0 5 10 50 100 500 1000 1500 2000
Abemaciclib (nM)
Syn. score: 6,7 Most syn. Area: 12,5
SK-N-SH
Dose-response matrix
2000 66 64 67 69 68 66 70 70 72
1500 54 58 59 61 61 63 66 65 67
1000 40 44 50 51 55 57 58 60 61
500 16 25 38 41 46 49 46 50 55
100 -2 11 21 29 33 37 42 44 45
0 0 11 22 31 35 38 42 45 45
0 100 500 1000 2000 3000 4000 5000 6000
Ribociclib (nM)
Syn. score: 6,9 Most syn. Area: 12,4
Dose-response matrix
2000 75 72 76 78 78 80 81 81 83
1500 62 65 65 69 71 76 77 76 78
1000 46 49 53 61 66 69 69 72 74
500 25 28 35 48 56 62 64 64 67
100 6 10 19 35 43 51 53 56 60
0 0 7 16 34 39 48 53 57 58
0 5 10 50 100 500 1000 1500 2000
Abemaciclib (nM)
Syn. score: 8,6 Most syn. Area: 16,4

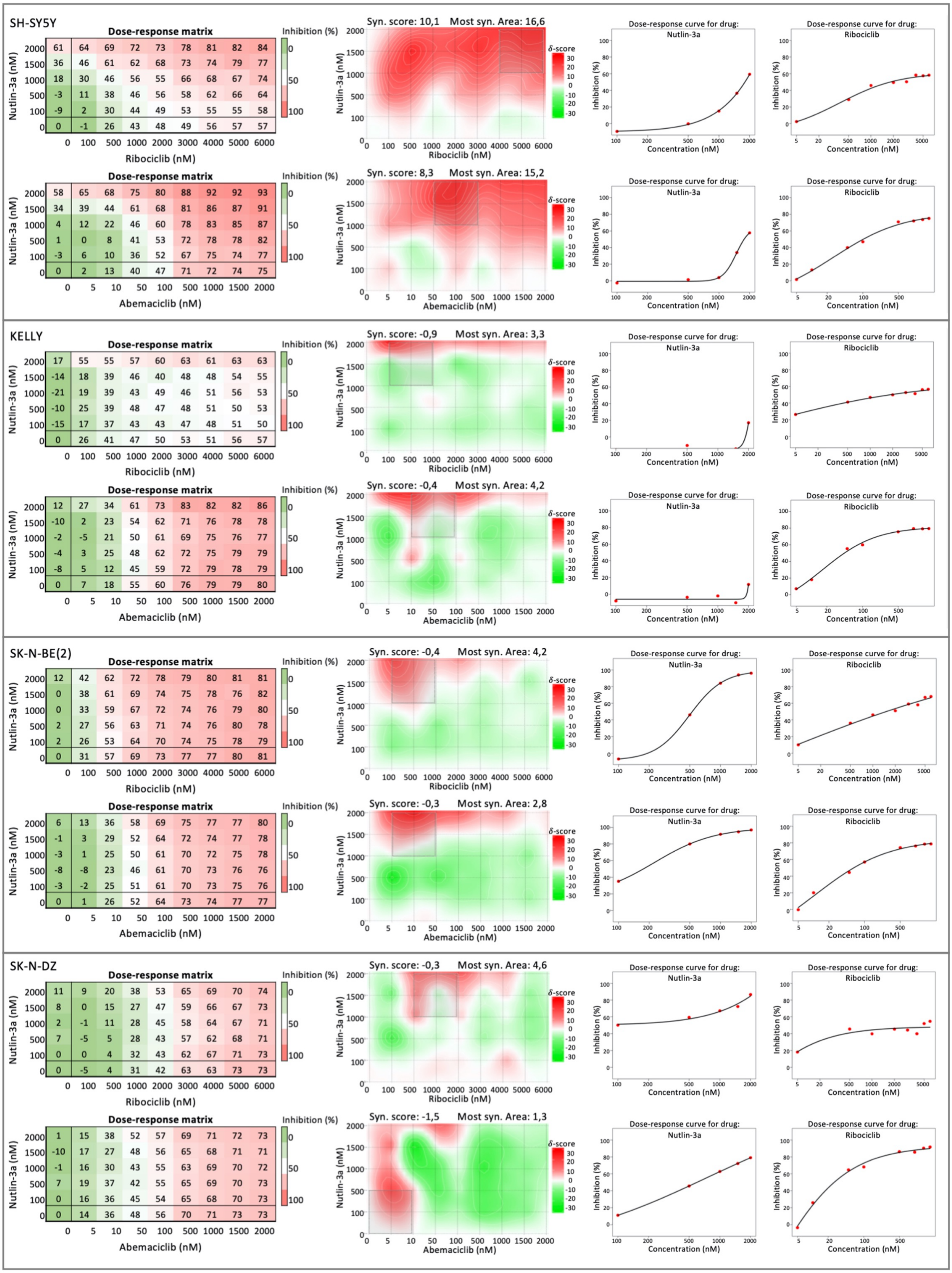

SH-SY5Y
Dose-response matrix
Nutlin-3a (nM)
Ribociclib (nM)
Inhibition (%)
Syn. score: 10,1 Most syn. Area: 16,6
Syn. score: 8,3 Most syn. Area: 15,2
Abemaciclib (nM)
δ-score
Dose-response curve for drug: Nutlin-3a
Dose-response curve for drug: Ribociclib
Inhibition (%)
Concentration (nM)
KELLY
Syn. score: -0,9 Most syn. Area: 3,3
Syn. score: -0,4 Most syn. Area: 4,2
SK-N-BE(2)
Syn. score: -0,4 Most syn. Area: 4,2
Syn. score: -0,3 Most syn. Area: 2,8
SK-N-DZ
Syn. score: -0,3 Most syn. Area: 4,6
Syn. score: -1,5 Most syn. Area: 1,3

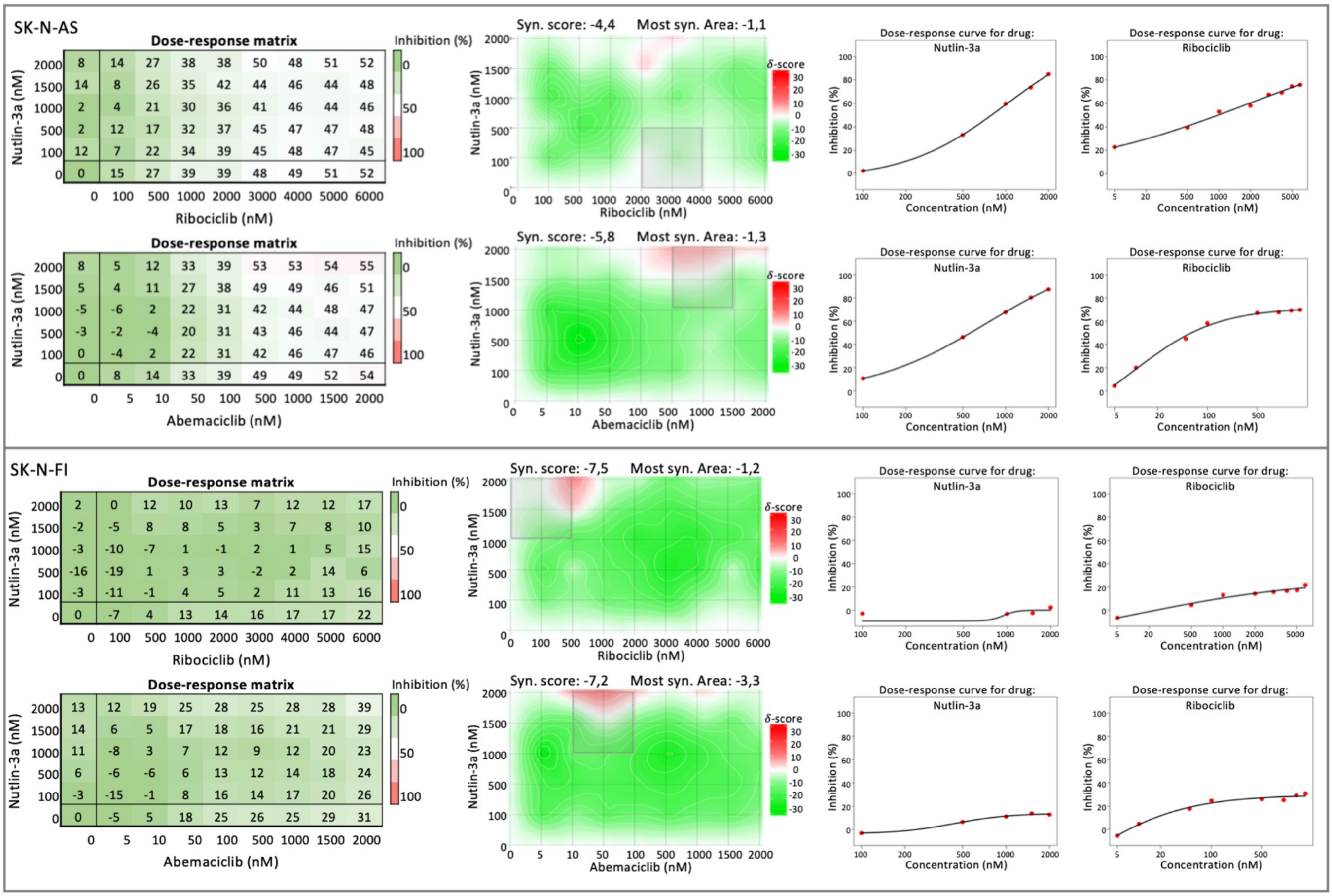

Supplemental Figure 7. Inhibition Dose-response matrix and synergy score heatmaps of neuroblastoma cell lines. Dose-response matrix, synergy score heatmaps and dose-response curves generated by SynergyFinder for combinational treatment with Nutlin-3a together with Ribociclib and Nutlin-3a together with Abemaciclib for all used cell lines using the viability data of the drug combination experiments.

Sup. Fig 8

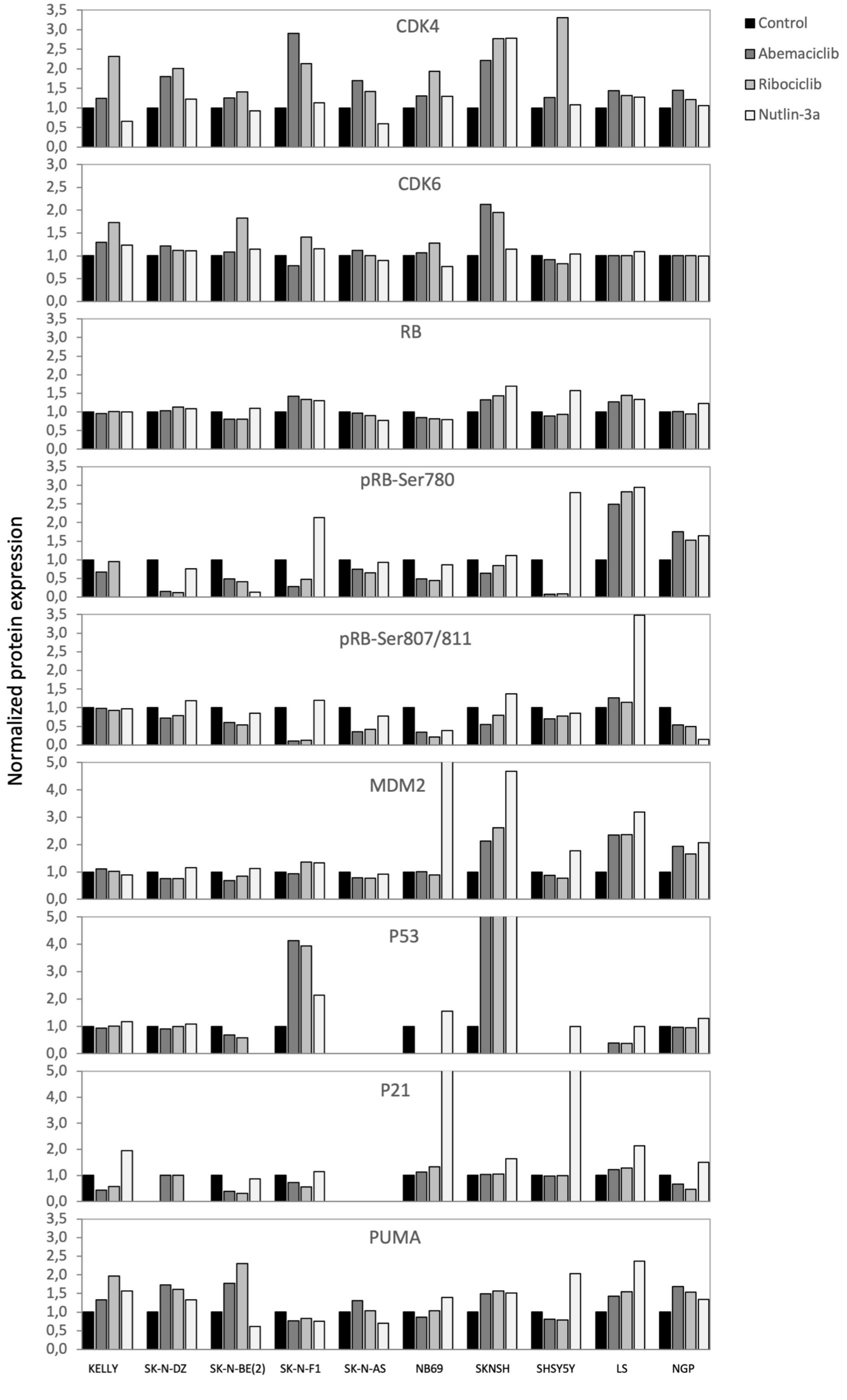

Supplemental Figure 8. Protein quantification of NB cell lines after CDK4i and MDM2i treatment. Graph representation of protein quantification of NB cells after Abemaciclib, Ribociclib and Nutlin-3a treatment. Quantification of the expression levels were performed by ImageJ and normalized against KU80 control.

Sup. Fig 9

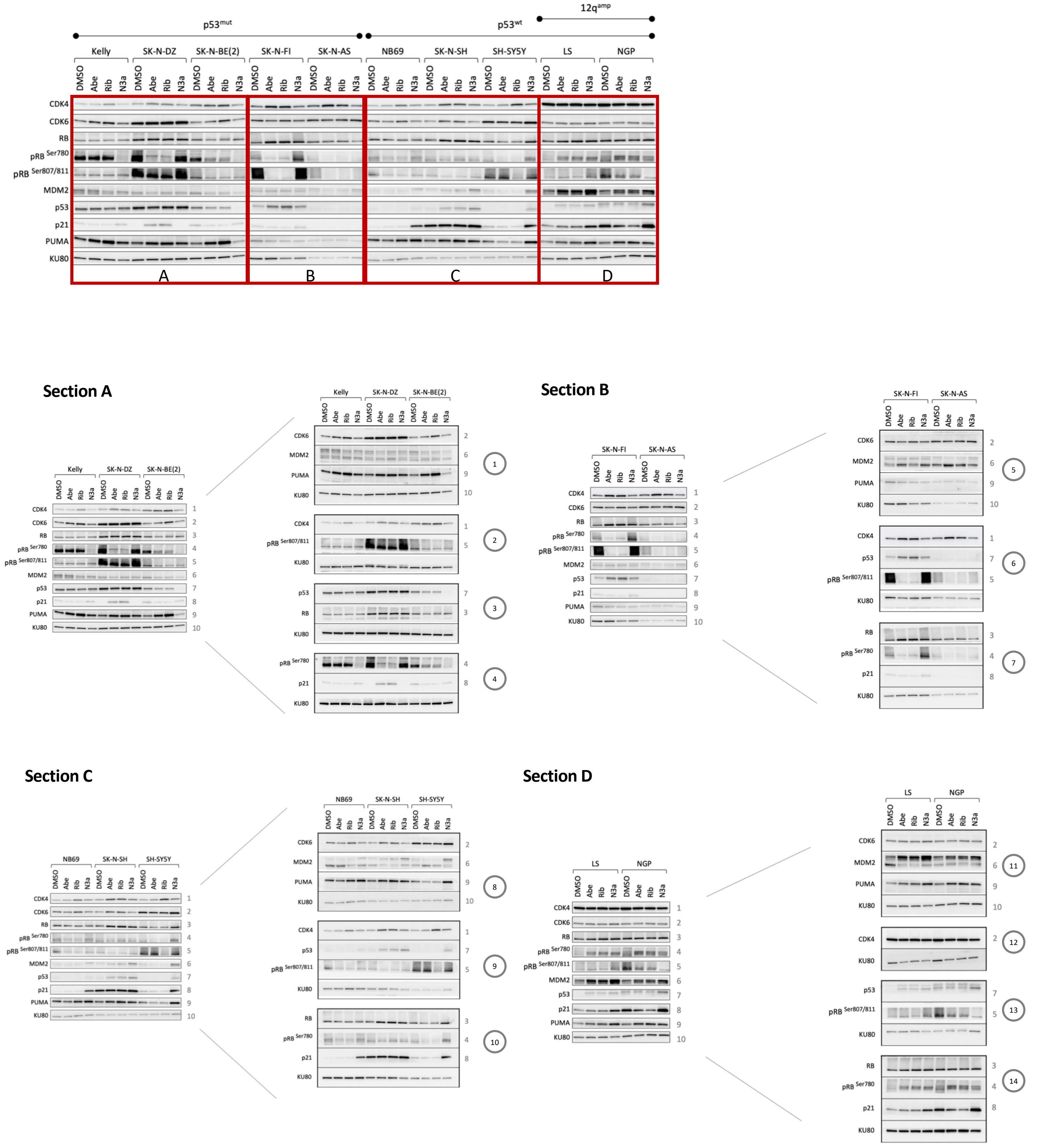

Supplemental Figure 9. Western blots for the CDK4 and MDM2 pathway analysis after the inhibition treatment. The blots were cut in multiple pieces according to size in order to optimize the number of proteins analyzed from a single membrane. All membranes used for Figure 6 have been numbered 1-14 as indicated by the circled numbers. Blot images for included pieces and respective KU80 control used for normalization are labelled according membrane number and shown for respective antibody. Blots processed in parallel but not included in the manuscript is marked by ”X”.

Sup. Fig 9, continued

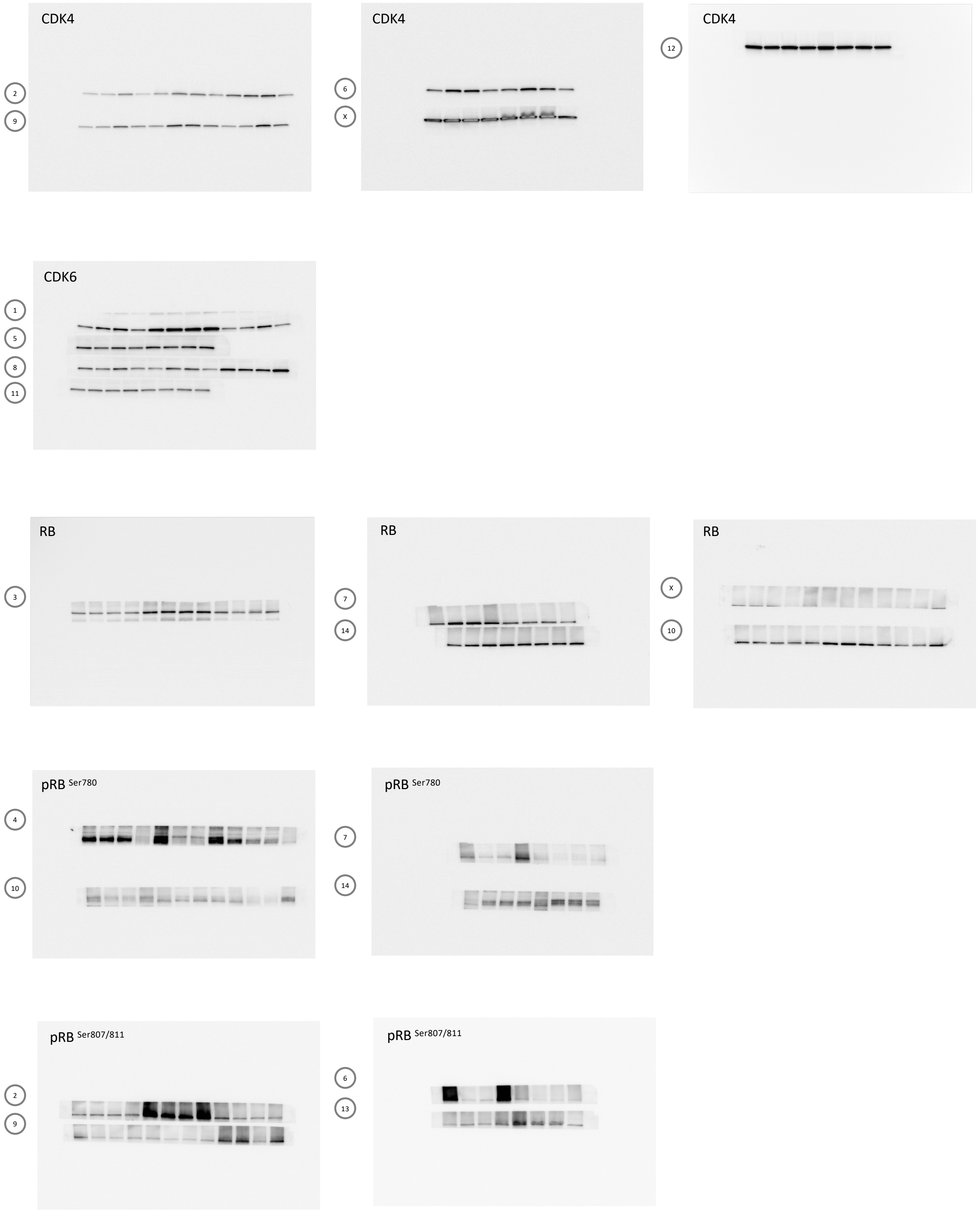

Sup. Fig 9, continued

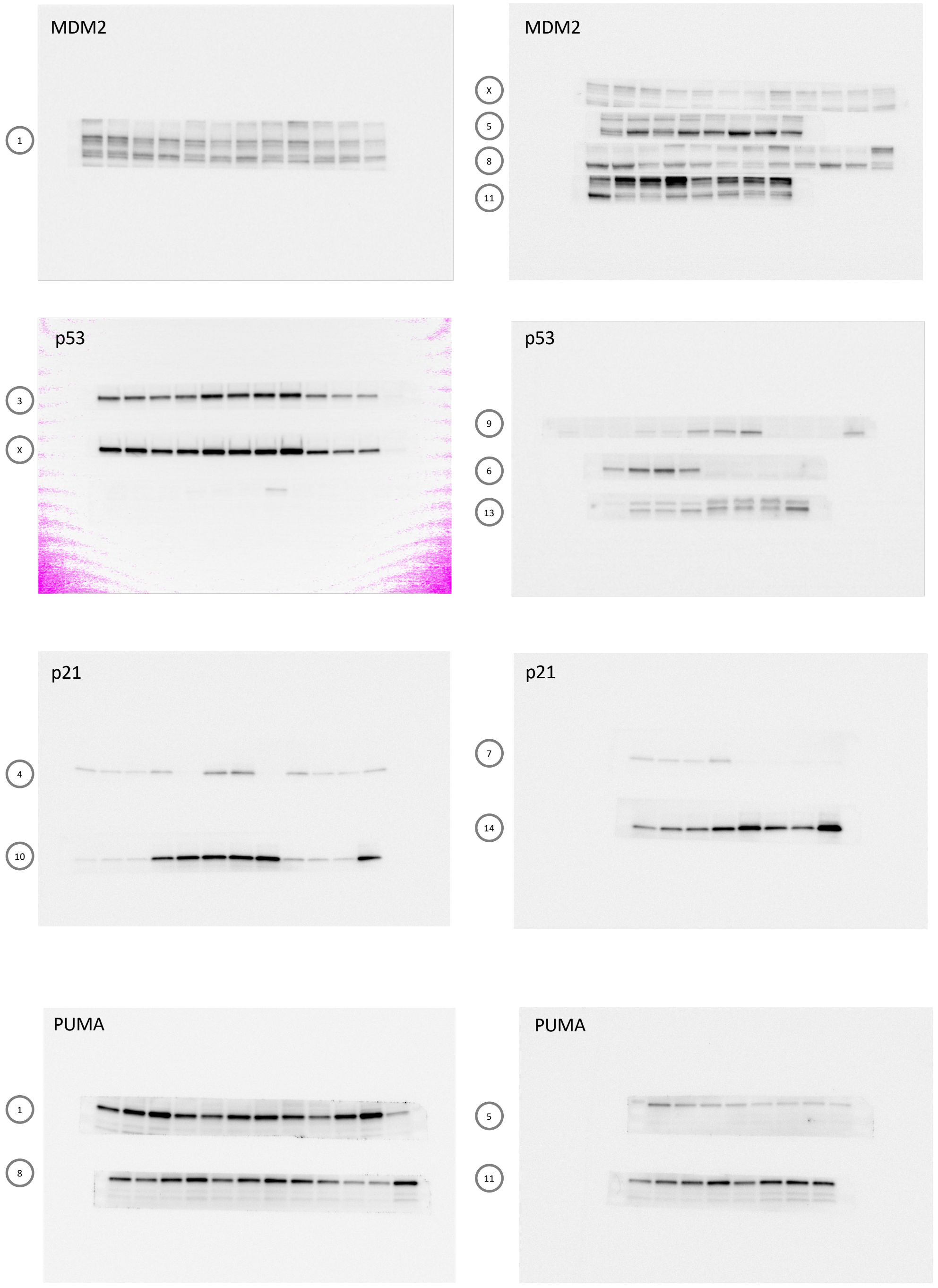

Sup. Fig 9, continued

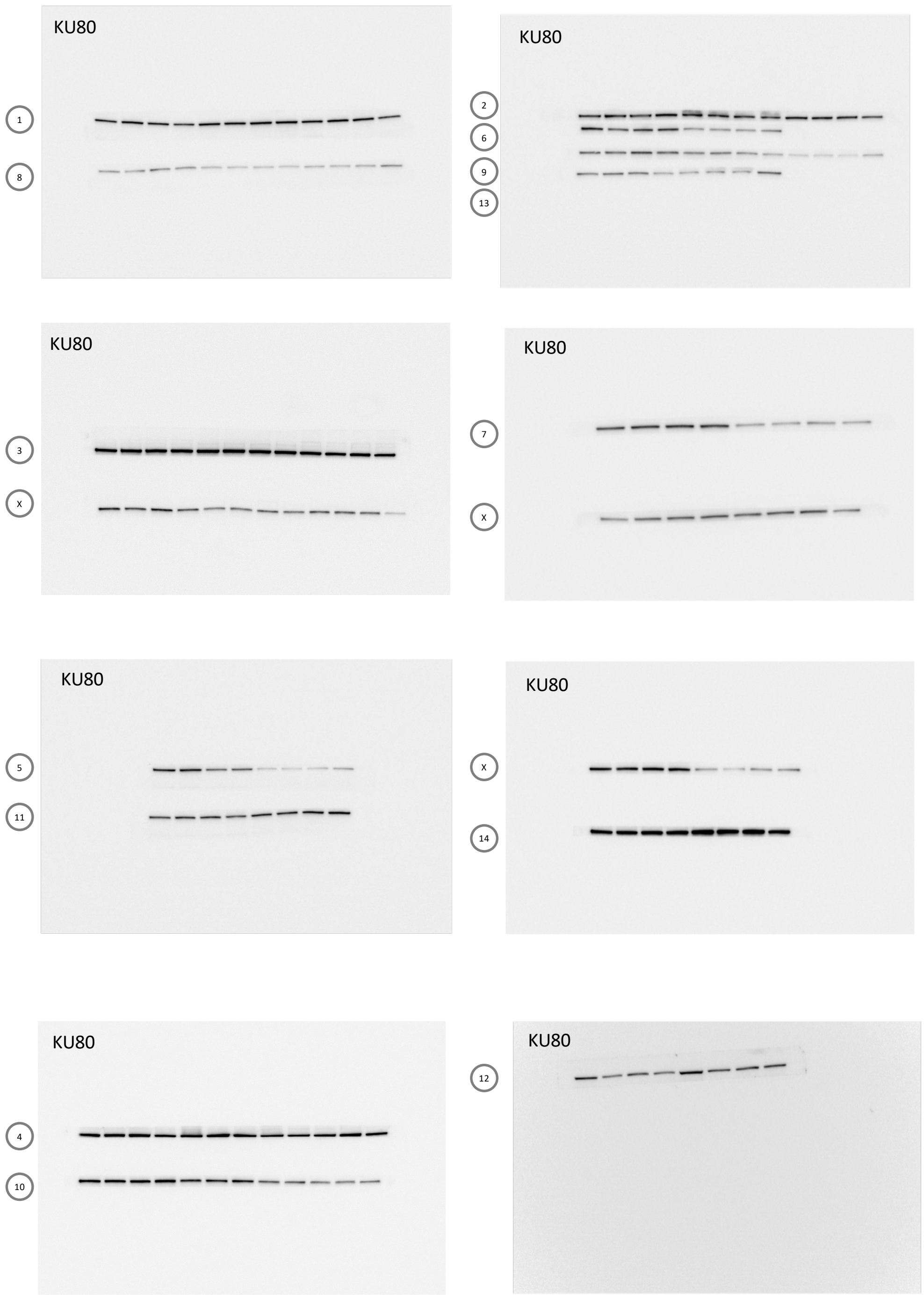

Sup. Fig 10

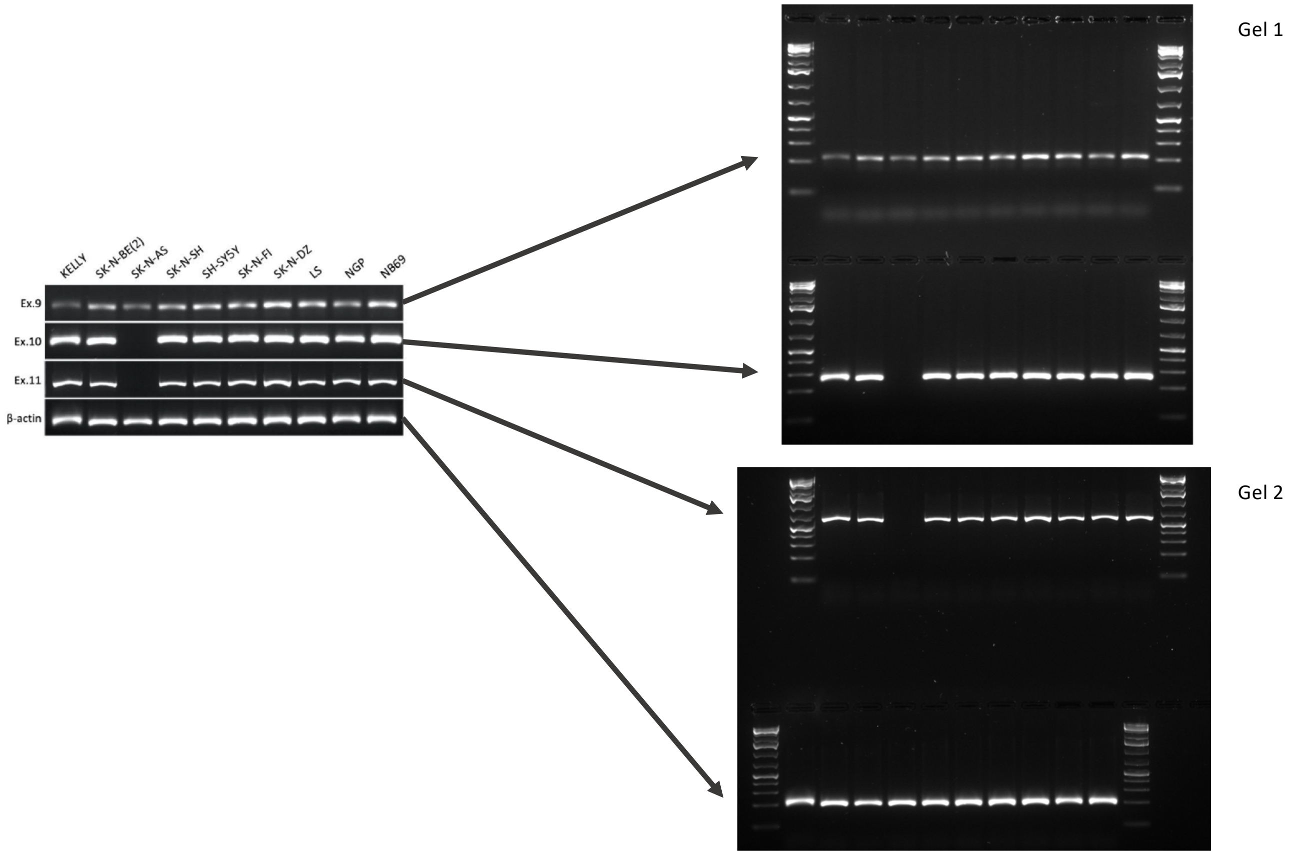

Supplemental Figure 10. Uncropped gel images corresponding to supplemental figure 4C.
